# Unconventional Hexagonal Close‐Packed High‐Entropy Alloy Surfaces Synergistically Accelerate Alkaline Hydrogen Evolution

**DOI:** 10.1002/advs.202409023

**Published:** 2024-11-08

**Authors:** Ting‐Hsin Hu, Cheng‐Yu Wu, Zong Ying He, Yi Chen, Liang‐Ching Hsu, Chih‐Wen Pao, Jui‐Tai Lin, Chun‐Wei Chang, Shang‐Cheng Lin, Rachel Osmundsen, Lee Casalena, Kun Han Lin, Shan Zhou, Tung‐Han Yang

**Affiliations:** ^1^ Department of Chemical Engineering National Tsing Hua University Hsinchu 300044 Taiwan; ^2^ College of Semiconductor Research National Tsing Hua University Hsinchu 300044 Taiwan; ^3^ Department of Soil and Environmental Sciences National Chung Hsing University Taichung 40227 Taiwan; ^4^ National Synchrotron Radiation Research Center Hsinchu 300092 Taiwan; ^5^ Thermo Fisher Scientific Hillsboro OR 97124 USA; ^6^ Department of Nanoscience and Biomedical Engineering South Dakota School of Mines and Technology Rapid City SD 57701 USA; ^7^ High Entropy Materials Center National Tsing Hua University Hsinchu 300044 Taiwan

**Keywords:** alkaline hydrogen evolution, atomic mixing, hexagonal close‐packed structure, high‐entropy alloy, operando X‐ray absorption spectroscopy

## Abstract

Accelerating the alkaline hydrogen evolution reaction (HER), which involves the slow cleavage of HO‐H bonds and the adsorption/desorption of hydrogen (H*) and hydroxyl (OH*) intermediates, requires developing catalysts with optimal binding strengths for these intermediates. Here, the unconventional hexagonal close‐packed (HCP) high‐entropy alloy (HEA) atomic layers are prepared composed of five platinum‐group metals to enhance the alkaline HER synergistically. The breakthrough is made by layer‐by‐layer heteroepitaxial deposition of subnanometer RuRhPdPtIr HEA layers on the HCP Ru seeds, despite the thermodynamic stability of Rh, Pd, Pt, and Ir in a face‐centered cubic (FCC) structure except for Ru. The synchrotron X‐ray absorption spectroscopy (XAS) confirms the atomic mixing and coordination environment of HCP RuRhPdPtIr HEA. Most importantly, they exhibit notable improvements in both electrocatalytic activity and durability for the HER in an alkaline environment, as compared to their FCC RuRhPdPtIr counterparts. Electrochemical measurements, *operando* XAS analysis, and density functional theory unveil that the binding strengths of H* and OH* intermediates on the active Pt and Ir sites can be weakened and strengthened to a moderate level, respectively, by mixing non‐active Ru, Rh, and Pd atoms with Pt and Ir atoms within the HCP HEA with strong synergistic electronic effects.

## Graphical abstract

Unconventional hexagonal close‐packed (HCP) high‐entropy alloy (HEA) surfaces are prepared to synergistically accelerate alkaline hydrogen evolution reaction (HER). Electrochemical measurements, X‐ray absorption spectroscopy, and density functional theory reveal optimized H* and OH* binding strengths on Pt and Ir sites for HER due to the synergistic electronic effects of HCP HEA.

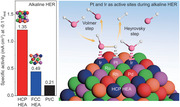

## 1 Introduction

Heterogeneous metal nanocatalysts hold immense importance in various industries and applications due to their exceptional catalytic performance in terms of activity, selectivity, and durability. These catalysts, typically composed of transition elements like platinum‐group metals (Ruthenium (Ru), Rhodium (Rh), Palladium (Pd), Platinum (Pt), and Iridium (Ir)), iron‐group metals (Iron (Fe), Cobalt (Co), and Nickel (Ni)), Gold (Au), Silver (Ag), and Copper (Cu) are pivotal in driving chemical reactions and processes forward with high efficiency. Very recently, diverse high‐entropy alloy (HEA) nanocatalysts consisting of at least five elements have been proven to exhibit excellent catalytic performance in many electrocatalytic and thermal catalytic reactions, achieving efficiencies that are beyond the capabilities of mono‐ and bi‐metallic nanocatalysts.^[^ ^1^, ^2^, ^3^, ^4^, ^5^, ^6^, ^7^, ^8^, ^9^, ^10^, ^11^, ^12^, ^13^, ^14^, ^15^, ^16^, ^17^, ^18^, ^19^, ^20^, ^21^, ^22^, ^23^ ^]^ The unique interplay between the multiple elements on the surface of HEA nanocatalysts and thus synergistic effect (often referred to as the cocktail effect) can create a diverse range of active sites with optimal binding energies for reactants, intermediates, and products.^[^ ^4^, ^8^, ^9^, ^10^, ^11^ ^]^ For example, recent theoretical calculations have validated a pronounced synergistic effect arising from the multi‐element composition of PtFeCoNiCu HEA nanoparticles.^[^ ^11^ ^]^ This effect demonstrates potential in fine‐tuning the free energy of hydrogen adsorption (ΔG_H*_), a commonly used descriptor for the efficiency of the hydrogen evolution reaction (HER). The optimal ΔG_H*_ value on the HEA surface is obtained by combining the strong hydrogen‐binding capabilities of Fe, Co, and Ni with the moderate hydrogen binding of Pt, as well as the weaker hydrogen binding of Cu, following the Sabatier principle. In addition, the high degree of disorder at the atomic level can even convert inactive sites into active ones for catalysis.^[^ ^12^ ^]^ Furthermore, HEA nanocatalysts often exhibit a decreased susceptibility to catalyst poisoning by contaminants or reactants during the catalysis, as well as exceptional durability to harsh reaction conditions owing to a high‐entropy atomic environment.^[^ ^13^, ^14^, ^15^, ^16^, ^17^, ^18^ ^]^

**Figure 1:**
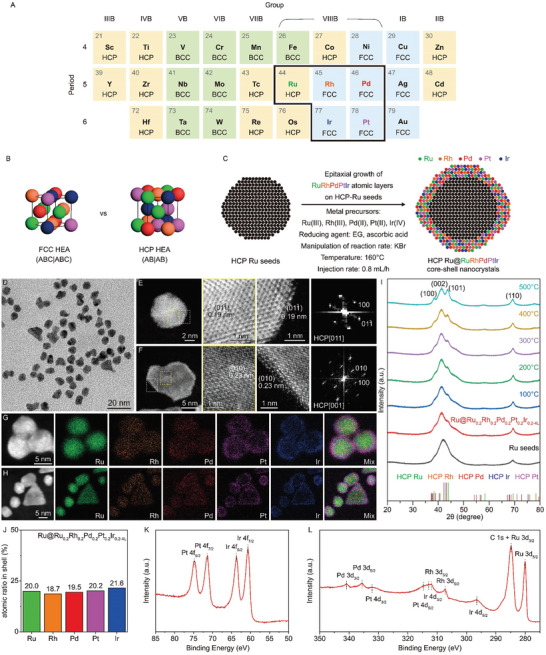
Synthetic design and characterization of HCP Ru@RuRhPdPtIr core‐shell nanocrystals. A) The thermodynamically stable crystal structures of transition metals (HCP highlighted in orange, FCC highlighted in blue, and BCC highlighted in green). B) The unit cells of FCC and HCP RuRhPdPtIr HEA. C) Schematic of epitaxial growth to obtain HCP RuRhPdPtIr shells on Ru seeds. D–H) TEM, HAADF‐STEM, FFT, and EDS mapping analysis of Ru@Ru_0.2_Rh_0.2_Pd_0.2_Pt_0.2_Ir_0.2‐4L_ core‐shell nanocrystals. I) Synchrotron HRPXRD analysis of Ru seeds and Ru@Ru_0.2_Rh_0.2_Pd_0.2_Pt_0.2_Ir_0.2‐4L_ with in situ heating. J) ICP‐OES analysis of Ru_0.2_Rh_0.2_Pd_0.2_Pt_0.2_Ir_0.2‐4L_ shell. K,L) XPS spectra of Ru@Ru_0.2_Rh_0.2_Pd_0.2_Pt_0.2_Ir_0.2‐4L_ in the regions of (K) 50–85 eV and (L) 275–350 eV.

While compositional control shows significant potential for catalyst discovery, current HEA nanocrystals are predominantly restricted to a face‐centered cubic (FCC) structure constructed by the close stacking in ABC|ABC sequence (**Figure** **1**A; and Table S1, Supporting Information).^[^ ^5^, ^6^, ^7^, ^8^, ^9^, ^10^, ^11^, ^12^, ^13^, ^14^, ^15^, ^16^, ^17^, ^18^, ^19^, ^20^, ^21^ ^]^ This limitation arises from the increased difficulty of achieving multi‐element atomic mixing, which is necessary for the formation of a solid‐solution HEA phase. As a result, synthetic strategies must be devised to facilitate high‐entropy random mixing, thereby expediting the inter‐diffusion of metal atoms and ultimately resulting in the formation of the FCC structure. The FCC structure aligns with the thermodynamically stable configurations of the principal constituent elements involved, especially since the majority of catalytic metals adopt the FCC structure. For example, octonary PtPdCoNiFeCuAuSn HEA nanocrystals featuring homogeneous mixing undergo crystallization in the FCC structure by thermally shocking metal precursor mixtures at a high temperature of ≈2000 K.^[^ ^18^ ^]^ For another example, the FCC structure of the quinary PtIrCuNiCr HEA nanocrystals is synthesized in nanoseconds using laser scanning ablation.^[^ ^19^ ^]^ Furthermore, through the tight control over the reduction kinetics of the mixed metal precursor solution during wet‐chemical synthesis at temperatures ≈500 K, the quinary PdPtIrRuRh and octonary PdPtIrRuRhOsAuAg HEA nanocrystals both show the polycrystalline FCC structure.^[^ ^10^, ^20^ ^]^ These pioneering studies highlight that current synthetic approaches predominantly yield HEA nanocrystals in their thermodynamically stable FCC structures. Despite the advances in synthesis, achieving alternative crystal structures is still difficult, limiting the exploration of other potentially beneficial configurations. How to not only engineer the crystal structures of HEA nanocrystals consisting of predominant transition elements into unconventional structures but also to achieve a significant level of multi‐element atomic mixing remains a great challenge. Given the well‐established superior electrocatalytic performance of hexagonal close‐packed (HCP)‐structured nanocatalysts in alkaline HER, as evidenced by previous studies,^[^ ^24^, ^25^, ^26^ ^]^ our investigation centers on an HCP HEA structure featuring the characteristic AB|AB stacking sequence (Figure 1B). The manipulation of crystal structure in HEA nanocrystals is anticipated to efficiently tune their properties due to the different synergistic effects arising from the distinct stacking sequences of atoms in the structure and atomic arrangements on the surface. It will offer an avenue for the rational synthesis of high‐performance HEA nanocatalysts for various catalytic applications.

In this work, we report the controlled synthesis of atomic‐mixing RuRhPdPtIr HEA atomic layers made of all platinum‐group metals into an unconventional HCP structure, although four constituent elements of Rh, Pd, Pt, and Ir are thermodynamically stable in a FCC structure except for Ru with a preferred HCP lattice (Figure 1A). Our breakthrough is made possible by robust layer‐by‐layer heteroepitaxial deposition of RuRhPdPtIr HEA atomic layers on the HCP Ru seeds through the dropwise addition of the mixed metal precursor mixtures in a polyol synthesis. Through synchrotron high‐resolution powder X‐ray diffraction (HRPXRD) with in situ heating, we demonstrate that the HCP structure of RuRhPdPtIr HEA is stable up to 500 °C. Importantly, HCP RuRhPdPtIr layers exhibit notable improvements in both electrocatalytic activity and durability for the HER in an alkaline environment, compared to their FCC RuRhPdPtIr HEA counterparts. This demonstrates that the HER catalytic performance is highly dependent on the crystal structure of HEA. Furthermore, electrochemical measurements, *operando* XAS analysis (XAS), and density functional theory (DFT) unveil strong synergistic electronic interactions among the five elements within the HCP RuRhPdPtIr during alkaline HER. These atomic‐scale interactions result in suitable H and OH binding strengths for HCP RuRhPdPtIr, with Pt and Ir active sites specifically undergoing H and OH adsorption, respectively, thereby contributing to the observed HER catalytic enhancement.

## 2 Results and Discussion

### 2.1 Synthesis and Characterizations of HCP and FCC HEA Structures

In this work, we synthesize atomic‐mixing RuRhPdPtIr*_n_*_L_ HEA shells with different numbers (*n*) of atomic layers on the HCP Ru seeds to replicate the structure of the seeds through the heteroepitaxial growth in the dropwise synthesis (Figure 1C). This heteroepitaxial growth is primarily governed by the relative rates of atomic deposition and atomic diffusion on the seed surface.^[^ ^27^, ^28^ ^]^ When atomic diffusion on the surface is faster than deposition, a “hit‐and‐run” mechanism allows the deposited atoms to migrate and organize themselves in a layer‐by‐layer growth mode. This results in a smooth, conformal shell, particularly when few atomic layers are deposited. We first prepare HCP Ru seeds through a polyol synthesis, resulting in products containing ≈94.5% spherical‐like nanocrystals with an average size of 6.7 nm, along with 5.5% plate‐like nanosheets (Figure S1, Supporting Information).^[^ ^29^, ^30^ ^]^ We then deposit equiatomic RuRhPdPtIr HEA alloys with ≈4 atomic layers on the HCP Ru seeds through the heteroepitaxial growth strategy for the fabrication of Ru@Ru_0.2_Rh_0.2_Pd_0.2_Pt_0.2_Ir_0.2‐4L_ core‐shell nanocrystals with an unconventional HCP structure. A precursor mixture of Ru(acac)_3_, Rh(acac)_3_, Pd(acac)_2_, Pt(acac)_2_, and H_2_IrCl_6_·xH_2_O in ethylene glycol (EG; solvent and reducing agent) is very slowly injected at a rate of 0.8 mL h^−1^ into another EG solution that contained Ru seeds, ascorbic acid (AA; reducing agent), potassium bromide (KBr; manipulation of reaction rate^[^ ^21^ ^]^), and polyvinylpyrrolidone (PVP; colloidal stabilizer) at a high temperature of 160 °C. In the case of Ir, H_2_IrCl_6_·xH_2_O is utilized as the precursor because of the slower reduction kinetics observed with Ir(acac)_3_ (Figure S2, Supporting Information). The gradual introduction of the precursor solution, drop by drop, enables precise control over the generation of atoms from the reduction of all five precursors, maintaining a nearly uniform pace for each.^[^ ^21^, ^31^ ^]^ The high diffusion rate relative to deposition allows for layer‐by‐layer growth. Consequently, it facilitates the epitaxial growth of RuRhPdPtIr onto the Ru seeds, resulting in an atomic‐mixing HEA phase with an unusual HCP structure.

Figure 1D shows the transmission electron microscopy (TEM) image of Ru_0.2_Rh_0.2_Pd_0.2_Pt_0.2_Ir_0.2‐4L_ HEA shells on the 6.7‐nm HCP Ru seeds. The size of the major nanocrystals with spherical‐like shape is increased to 8.4 nm. Given that the total thickness of the HEA shells is ≈1.7 nm, with each side contributing ≈0.85 nm, this corresponds to ≈4 atomic layers. To further observe the atomic arrangement, the obtained Ru@Ru_0.2_Rh_0.2_Pd_0.2_Pt_0.2_Ir_0.2‐4L_ core‐shell nanocrystals are characterized by the high‐angle annular dark‐field scanning transmission electron microscopy (HAADF‐STEM) with atomic resolution (Figure 1E,F). A contrast is observed between the Ru core and the outer RuRhPdPtIr surface composed of a few atomic layers, which can be attributed to the difference in atomic number among the elements. For the individual spherical‐like nanocrystal, the periodic lattice extending across the whole nanocrystal reveals epitaxial growth mode. The corresponding fast Fourier transform (FFT) pattern shows only one set of an HCP‐diffraction pattern along the [011] zone axis of an HCP structure. The lattice spacing of (01$\bar{1}$) planes for both the Ru cores and RuRhPdPtIr shells are identical, measuring ≈0.19 nm. The atomic‐resolution HAADF‐STEM image and the corresponding FFT pattern reveal that the individual sheet‐like nanocrystal retains the characteristic hexagonal atomic arrangement when observed along the HCP [001] zone axis. The lattice distance for the Ru cores and the RuRhPdPtIr (010) plane is measured to be ≈0.23 nm. These observations highlight the preservation of the HCP structure in the Ru core and HEA shell, with precise atomic alignment and lattice matching.

The energy‐dispersive X‐ray spectrometry (EDS) elemental mappings confirm the random and homogeneous distributions of five elements on the Ru cores (Figure 1G,H). In addition, the 3D EDS tomography further reveals the spatial elemental distributions of Ru cores and RuRhPdPtIr shells (Video S1, Supporting Information). The atomic percentages of the Ru, Rh, Pd, Pt, and Ir elements of the HEA shells are determined to be 20%, 18.7%, 19.5%, 20.2%, and 21.6% through the inductively coupled plasma optical emission spectrometry (ICP‐OES), respectively (Figure 1J). Again, the average number of HEA atomic layers on the Ru seeds is estimated to be ≈4 atomic layers, which is derived from the atomic percentages in the nanocrystals, with the core constituting 51.2% and the shell 48.8%, as determined by ICP‐OES and quantitative analysis (Table S2, Supporting Information). This estimation aligns well with the observation of a HEA thickness of ≈1.7 nm in the TEM image. On the other hand, the surface chemical states of Ru@Ru_0.2_Rh_0.2_Pd_0.2_Pt_0.2_Ir_0.2‐4L_ core‐shell nanocrystals are characterized by X‐ray photoelectron spectroscopy (XPS) (Figure 1K,L).^[^ ^32^ ^]^ The doublets of the 3d orbitals of Ru, Rh, and Pd are observed in the 275–350 eV range, while the 4f orbitals of Pt and Ir are detected in the 50–85 eV range. The HCP HEA shells are primarily in the metallic state, with a small portion existing in the oxidized state, as evidenced by the binding energy values listed in Table S3 (Supporting Information) and the XPS fitting results (Figure S3, Supporting Information). To further expand the compositional diversity of HCP HEA, Ag, Fe, and Ni elements are successfully incorporated to synthesize octonary HCP RuRhPdPtIrAgFeNi HEA shells on Ru seeds (Figure S4, Supporting Information). This is achieved through heteroepitaxial growth in a dropwise synthesis. In this synthesis, the solvent is changed to triethylene glycol (TEG) instead of EG, and the reaction temperature is increased from 160 to 240 °C due to the lower reduction potentials of Fe(III) and Ni(II) precursors used. This demonstration indicates that the epitaxial engineering strategy is scalable.

The thermal stability of the Ru@Ru_0.2_Rh_0.2_Pd_0.2_Pt_0.2_Ir_0.2‐4L_ core‐shell nanocrystals is examined using synchrotron HRPXRD with in situ heating under vacuum conditions. Figure 1I displays the diffraction patterns collected at various temperatures, ranging from room temperature to 500 °C. For comparison, the corresponding Ru seeds are also presented in Figure S5 (Supporting Information). At room temperature, it can be observed that the two small peaks ≈38.5° and 43.9° appear after the epitaxial growth of RuRhPdPtIr HEA compared to Ru seeds, corresponding to HCP‐(100) and HCP‐(101) planes, respectively. However, the major characteristic peaks of the HCP (002) and (110) planes show no significant shift in either sample, consistent with the HAADF‐STEM results. As the temperature rises, the intensities of these HCP characteristic peaks increase in both the Ru seeds and Ru@Ru_0.2_Rh_0.2_Pd_0.2_Pt_0.2_Ir_0.2‐4L_ samples. This suggests an improvement in crystallinity at elevated temperatures. Importantly, no characteristic peaks of the FCC structure are observed, indicating the remarkable thermal stability of the unconventional HCP structure of the RuRhPdPtIr HEA on the Ru seeds up to 500 °C.

**Figure 2:**
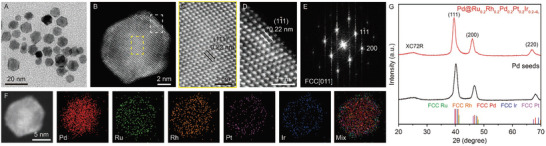
Characterization of FCC Pd@Ru_0.2_Rh_0.2_Pd_0.2_Pt_0.2_Ir_0.2‐4L_ core‐shell nanocrystals. A) TEM image. B) HAADF‐STEM image, enlarged HAADF‐STEM images for the C) Pd core and D) HEA shell, E) FFT pattern, and F) EDS mappings of (B). G) XRD patterns of Pd@Ru_0.2_Rh_0.2_Pd_0.2_Pt_0.2_Ir_0.2‐4L_ and Pd seeds dispersed on Vulcan XC72R carbon black.

As a control experiment, the crystal structure of RuRhPdPtIr HEA with ≈4 atomic layers can be tuned to FCC structure when we switch to the use of FCC spherical‐like Pd seeds with an average size of 7.70 nm (Figure S6, Supporting Information). The increase in the size of the obtained nanocrystals to ≈9.29 nm after growth suggests that the HEA shells comprise ≈4 atomic layers, as shown in Figure S6 (Supporting Information). **Figure** **2** shows the TEM, HAADF‐STEM images, FFT pattern, EDS mappings, and XRD analysis of Pd@Ru_0.2_Rh_0.2_Pd_0.2_Pt_0.2_Ir_0.2‐4L_ core‐shell nanocrystals, confirming the formation of conformal HEA shells. The specific atomic arrangement extending across the core‐shell structure is confirmed in the enlarged atomic‐resolution HAADF‐STEM image and FFT pattern, revealing that the Pd@Ru_0.2_Rh_0.2_Pd_0.2_Pt_0.2_Ir_0.2‐4L_ core‐shell nanocrystals adopt an FCC structure (Figure 2B–D). The lattice spacing of FCC‐(1$\bar{1}$1) plane is ≈0.22 nm. Additionally, the XRD pattern in Figure 2G shows two notable peaks at 2θ = 39.5° and 45.9°, corresponding to FCC (111) and (200) planes, respectively. The EDS mappings (Figure 2F) validate that these shells consist of deposited five PGMs in a solid‐solution phase.

**Figure 3:**
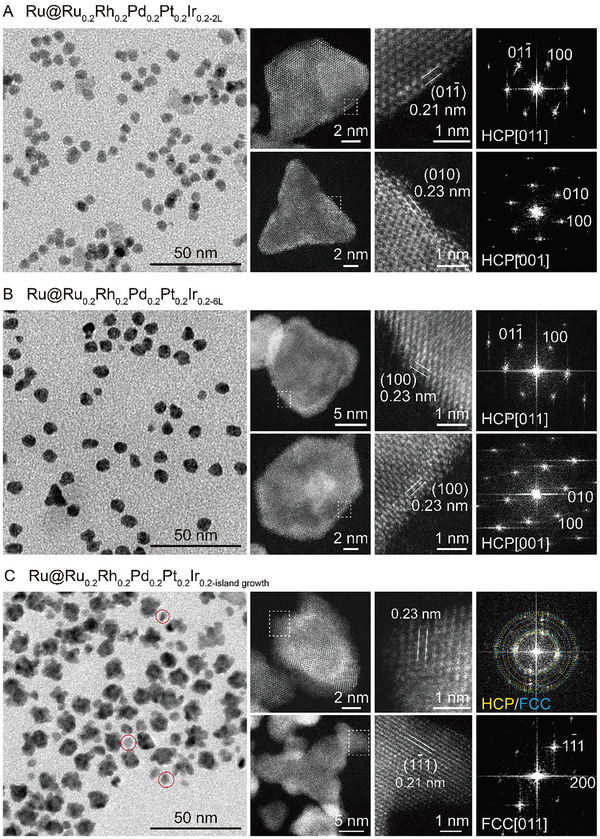
TEM, HAADF‐STEM, and FFT patterns of Ru@Ru_0.2_Rh_0.2_Pd_0.2_Pt_0.2_Ir_0.2_ core‐shell nanocrystals with different shell thickness. A) 2 atomic layers of shell. B) 6 atomic layers of shell. C) Island growth of shell (self‐nucleation particles are marked by red circles).

Furthermore, we explore how varying the thickness of the HEA shells impacts the crystal structure adopted by the HEA shells. In this approach, the HCP structure of the Ru core serves as a template for the Ru_0.2_Rh_0.2_Pd_0.2_Pt_0.2_Ir_0.2‐nL_ HEA shell during the growth. When the HEA shell is thin, the shell can adopt the same HCP structure as the Ru core through layer‐by‐layer epitaxial growth under the proper synthetic conditions (Figure 1). In addition, the strain also can be generated within the shell due to the mismatch (1.33% on *a* axis and 5.76% on *c* axis, Table S4, Supporting Information) in the atomic arrangement between the Ru core and the HEA shell. When the HEA shell grows thicker, the templating and strain effects induced at the core‐shell interface will be diminished since the atoms in the shell have more flexibility as they are farther from the core. Therefore, it is anticipated that the transition from the nonconventional HCP structure to the thermodynamically more stable FCC structure will occur as the shell thickens. In this study, the number of HEA atomic shells can be controlled to ≈2, 4, 6, and more than 6 by adjusting the concentration of the precursor mixture, as shown in **Figure** **3**. The core‐shell nanocrystals containing different numbers of HEA atomic layers are also confirmed by TEM, HAADF‐STEM images, and FFT patterns. The corresponding EDS mappings are provided in Figures S7–S9 (Supporting Information). When the number of HEA atomic layers is below 4 (Figure 1 and 3A), the smooth surfaces of the HEA shells on the Ru seeds are observed. These observations suggest the epitaxial growth of HEA and thus the HCP structure. As the number of atomic layers increases to ≈6 (Figure 3B), the morphology of the final products exhibits slight roughness, implying a gradual shift from epitaxial growth toward island growth. With the increase in the number of atomic layers exceeding 6 (i.e., Ru@Ru_0.2_Rh_0.2_Pd_0.2_Pt_0.2_Ir_0.2‐island growth_), significantly rough surfaces are observed on both spherical and sheet‐like nanocrystals (Figure 3C). As a result, the growth mode of the HEA shells on the Ru seeds is completely switched from layer‐by‐layer to island growth. In addition, a small portion of tiny particles of ≈3–5 nm is formed in the products, which can be ascribed to self‐nucleation. Most importantly, the HAADF‐STEM images and relevant FFT patterns taken from the HEA shells reveal the formation of mixed FCC/HCP and pure FCC structures, consistent with the synchrotron wide‐angle X‐ray scattering (WAXS) analysis (Figure S10, Supporting Information). The unconventional HCP structure of Ru_0.2_Rh_0.2_Pd_0.2_Pt_0.2_Ir_0.2_ HEA can be maintained within ≈6 atomic layers but shift to the thermodynamically stable FCC structure as the thickness increases further. This shift from layer‐by‐layer to island growth can be attributed to the diminishing influence of the templating effect of Ru seeds as the shell thickness increases, allowing the atoms in the shell greater flexibility to deviate from epitaxial alignment. Therefore, to achieve HCP HEA structures, it is crucial to ensure that the rate of atomic diffusion remains higher than the rate of deposition. Additionally, maximizing the templating effect of Ru seeds is key to maintaining the desired structure, especially during the early stages of shell formation.

### 2.2 Electronic Interactions and Atomic Mixing of HCP and FCC HEA Structures

**Figure 4:**
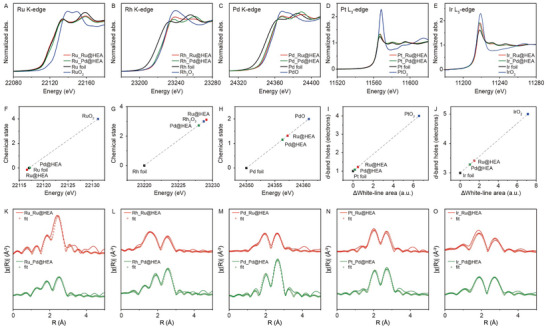
Electronic structures and atomic coordination environments of HCP Ru@Ru_0.2_Rh_0.2_Pd_0.2_Pt_0.2_Ir_0.2‐4L_ and FCC Pd@Ru_0.2_Rh_0.2_Pd_0.2_Pt_0.2_Ir_0.2‐4L_ core−shell nanocrystals. A–E) XANES spectra of HCP Ru@Ru_0.2_Rh_0.2_Pd_0.2_Pt_0.2_Ir_0.2‐4L_, FCC Pd@Ru_0.2_Rh_0.2_Pd_0.2_Pt_0.2_Ir_0.2‐4L_, and their corresponding metallic foils and oxidation states at the (A) Ru K‐edge, (B) Rh K‐edge, (C) Pd K‐edge, (D) Pt L_3_‐edge, and (E) Ir L_3_‐edge. (F‐J) Determination of chemical states and formal *d‐*band hole count of Ru, Rh, Pd, Pt, and Ir elements for HCP Ru@Ru_0.2_Rh_0.2_Pd_0.2_Pt_0.2_Ir_0.2‐4L_ and FCC Pd@Ru_0.2_Rh_0.2_Pd_0.2_Pt_0.2_Ir_0.2‐4L_: F–H) Absorption energy position versus chemical state of (F) Ru K‐edge, (G) Rh K‐edge, and (H) Pd K‐edge; I,J) White‐line peak area difference versus formal *d‐*band hole count of (I) Pt L_3_‐edge and (J) Ir L_3_‐edge. K–O) FT‐EXAFS spectra (lines) and curve fits (points) of HCP Ru@Ru_0.2_Rh_0.2_Pd_0.2_Pt_0.2_Ir_0.2‐4L_ and FCC Pd@Ru_0.2_Rh_0.2_Pd_0.2_Pt_0.2_Ir_0.2‐4L_ at the (K) Ru K‐edge, (L) Rh K‐edge, (M) Pd K‐edge, (N) Pt L_3_‐edge, and (O) Ir L_3_‐edge. The data are k^2^‐weighted and without phase correction.

The HCP and FCC structures of HEA atomic layers are anticipated to induce distinct synergistic electronic effects owing to the different stacking sequences of atoms within the structure and the arrangements of atoms on the surface. Therefore, we employ synchrotron XAS analysis to probe the electronic interactions between the elements and further reveal their atomic details in both the HCP Ru@Ru_0.2_Rh_0.2_Pd_0.2_Pt_0.2_Ir_0.2‐4L_ and FCC Pd@Ru_0.2_Rh_0.2_Pd_0.2_Pt_0.2_Ir_0.2‐4L_ core‐shell nanocrystals, each possessing a shell thickness of ≈4 atomic layers. A comparison of X‐ray absorption near‐edge structure (XANES) spectra for all elements in both HCP Ru@Ru_0.2_Rh_0.2_Pd_0.2_Pt_0.2_Ir_0.2‐4L_ and FCC Pd@Ru_0.2_Rh_0.2_Pd_0.2_Pt_0.2_Ir_0.2‐4L_ samples, along with their metallic foils and oxides, is shown in **Figure** **4**A–E. The four samples exhibit minor variations in their absorption edge and post‐edge regions, evident in differences in shape, intensity, and oscillation patterns. These deviations likely suggest orbital hybridizations among the mixed elements, indicating changes in the electronic structure. To further investigate the chemical states of Ru, Rh, and Pd elements in the HEA samples, we determine their absorption K‐edge positions by identifying the inflection points of the pre‐edges (Figure S11, Supporting Information). Subsequently, we derive their chemical states by establishing a linear relationship between absorption edge positions and the chemical states of control samples including metallic foils and oxides (Figure 4F–H).^[^ ^33^ ^]^ Note that in the XAS spectra, the Ru signal should be contributed from both Ru cores and Ru in the HEA shells for the HCP HEA sample, while the Pd signal should originate from both Pd cores and Pd in the HEA shells for the FCC HEA sample. Additionally, the electronegativities of Ru and Pd are both 2.20, implying that the difference in electronic properties should be attributed to the distinct atomic arrangements of HCP and FCC structures rather than the different core materials. For Ru, the average valence states in both HCP and FCC HEA structures are ≈0. However, Rh and Pd exhibit higher valence states in the HCP HEA sample compared to the FCC HEA sample. Surprisingly, for Rh, the average valence state in the HCP HEA sample is even slightly higher than that in the reference sample of Rh_2_O_3_, suggesting electron transfer from Rh to neighboring atoms in the high‐entropy environment. For Pd, the average valence state of Pd is in this sequence: Pd foil (Pd^0^) < Pd in FCC < Pd in HCP < PdO (Pd^2+^). On the other hand, we also examine the chemical states of Pt and Ir in the HEA samples, along with their formal *d*‐band hole count, by integrating the area of the white‐line peak in the L_3_‐edge XANES spectra (Figure 4I,J; Figure S12, Supporting Information).^[^ ^34^, ^35^, ^36^, ^37^ ^]^ For Pt, the valence states in both the HCP and FCC HEA structures show comparable chemical states that are near the metallic state. For Ir, the valence states of Ir in the HCP and FCC HEA structures follow this trend: Ir foil (Ir^0^) < Ir in FCC < Ir in HCP < IrO_2_ (Ir^4+^). The above quantitative information suggests that there are distinct electronic interactions between the mixed elements present in the HCP and FCC HEA structures.

Furthermore, the Fourier‐transformed extended X‐ray absorption fine structure (FT‐EXAFS) of the HCP Ru@Ru_0.2_Rh_0.2_Pd_0.2_Pt_0.2_Ir_0.2‐4L_ and FCC Pd@Ru_0.2_Rh_0.2_Pd_0.2_Pt_0.2_Ir_0.2‐4L_ core‐shell nanocrystals shows alloy doublet peaks between R = 1.8 and 2.7 Å for all elements compared to their metallic foils, as shown in Figure 4K–O and Figure S13 (Supporting Information). These observations can be attributed to the atomic interactions occurring between the constituent elements.^[^ ^38^, ^39^, ^40^, ^41^ ^]^ The FT‐EXAFS spectra are fitted to further elucidate the coordination environment of HCP Ru@Ru_0.2_Rh_0.2_Pd_0.2_Pt_0.2_Ir_0.2‐4L_ and FCC Pd@Ru_0.2_Rh_0.2_Pd_0.2_Pt_0.2_Ir_0.2‐4L_, along with their respective seeds and the metallic foils for each element (Figure 4K–O; Figures S14 and S15, Supporting Information and **Table** **1**). Based on the fitting results for Ru@Ru_0.2_Rh_0.2_Pd_0.2_Pt_0.2_Ir_0.2‐4L_, the coordination values of M‐4d (Ru, Rh, and Pd)/M‐5d (Pt, Ir) for Rh, Pd, Pt, and Ir are determined to be 3.22/3.01, 3.10/4.35, 3.45/4.49, and 3.77/4.98, respectively. The radial distances of Pt‐5d (2.713 Å) and Ir‐5d (2.607 Å) within the HCP HEA atomic layers are shorter than the Pt‐Pt (2.750 Å) and Ir‐Ir (2.706 Å) distances in the corresponding Pt and Ir foils. In addition, the coordination numbers of M‐4d and M‐5d bonds are all significantly higher than those of M‐O bonds, confirming that these elements primarily exist in their metallic states. These findings suggest that 4d and 5d elements are capable of forming bonds with each other, indicating the presence of a high‐entropy atomic‐mixing phase instead of elemental segregation. For the Ru, an increased ratio of 6.52/2.05 is confirmed, which can be ascribed to the XAS signals originating from the Ru cores and HEA shells, highlighting a significant presence of Ru‐Ru bonding. A similar phenomenon is observed for Pd in the case of FCC Ru_0.2_Rh_0.2_Pd_0.2_Pt_0.2_Ir_0.2‐4L_ on the Pd seeds. Moreover, the wavelet analysis of the k^2^‐weighted EXAFS (WT‐EXAFS) demonstrates a significant difference in coordination environments between HCP and FCC HEA structures as well as their corresponding metallic foils and oxides (Figure S16, Supporting Information), indicating that each element is surrounded by distinct species. Together, the above XAS measurements not only reveal distinct electronic interactions in the HCP and FCC HEA structures but also identify their high‐entropy atomic‐mixing phases.

**Table 1:** The EXAFS fitting results of Ru@Ru_0.2_Rh_0.2_Pd_0.2_Pt_0.2_Ir_0.2‐4L_, Pd@Ru_0.2_Rh_0.2_Pd_0.2_Pt_0.2_Ir_0.2‐4L_, Ru seeds, Pd seeds, and the corresponding foils.

Sample^a)^	Bond	CN^b)^	R^c)^ [Å]	σ^2^ ^d)^	S_0_^2^ ^e)^	ΔE_0_ ^f)^ [eV]	R‐factor^g)^
Ru foil	Ru‐Ru	12	2.653	0.00300	0.701	−1.008	0.041
Ru seeds	Ru‐Ru	9.20	2.661	0.00456			
Ru_Ru@HEA	Ru‐5d	2.05	2.855	0.00634			
	Ru‐4d	6.52	2.706	0.00302			
Ru_Pd@HEA	Ru‐5d	3.09	2.768	0.00814			
	Ru‐4d	4.11	2.737	0.00339			
	Ru‐O	1.46	2.018	0.00354			
Rh foil	Rh‐Rh	12	2.685	0.00323	0.768	−2.029	0.023
Rh_Ru@HEA	Rh‐5d	3.01	2.721	0.00798			
	Rh‐4d	3.22	2.716	0.00628			
	Rh‐O	2.57	2.027	0.00313			
Rh_Pd@HEA	Rh‐5d	3.34	2.744	0.00584			
	Rh‐4d	4.00	2.741	0.00315			
	Rh‐O	1.37	2.029	0.00788			
Pd foil	Pd‐Pd	12	2.749	0.00501	0.765	1.062	0.018
Pd seeds	Pd‐Pd	9.10	2.737	0.00534			
Pd_Ru@HEA	Pd‐5d	4.35	2.694	0.00364			
	Pd‐4d	3.10	2.675	0.00302			
	Pd‐O	0.80	1.983	0.00467			
Pd_Pd@HEA	Pd‐5d	3.18	2.769	0.00570			
	Pd‐4d	6.98	2.762	0.00407			
Pt foil	Pt‐Pt	12	2.750	0.00390	0.757	4.637	0.023
Pt_Ru@HEA	Pt‐5d	4.49	2.713	0.00388			
	Pt‐4d	3.45	2.694	0.00303			
	Pt‐O	0.51	1.988	0.00337			
Pt_Pd@HEA	Pt‐5d	4.49	2.720	0.00304			
	Pt‐4d	4.72	2.708	0.00416			
Ir foil	Ir‐Ir	12	2.706	0.00358	0.809	0.792	0.025
Ir_Ru@HEA	Ir‐5d	4.98	2.607	0.00304			
	Ir‐4d	3.77	2.573	0.00817			
Ir_Pd@HEA	Ir‐5d	4.47	2.685	0.00429			
	Ir‐4d	4.96	2.659	0.00587			

^a)^ The data fittings are conducted in the k range from 3 to 10 Å^−1^ for Ru, Rh, Pd, and Pt within Ru@Ru_0.2_Rh_0.2_Pd_0.2_Pt_0.2_Ir_0.2‐4L_, Pd@Ru_0.2_Rh_0.2_Pd_0.2_Pt_0.2_Ir_0.2‐4L_, Ru seeds, and Pd seeds. For Ru, Rh, Pd, and Pt foils, the fitted k range is from 3 to 11.5 Å^−1^. For Ir, the fitted k range is from 3 to 8.5 Å^−1^. Furthermore, all the elements are fitted in the R range from 1 to 3 Å;

^b)^ CN = coordination number;

^c)^ R = radial distance;

^d)^ σ^2^ = mean square relative displacements;

^e)^ S_0_^2^ = passive electron reduction factor;

^f)^ ΔE_0_ = fitted energy shift;

^g)^ R‐factor = Σ(data‐fit)^2^/Σdata^2^.

### 2.3 Electrocatalytic HER Performance: HCP versus FCC HEA Structures

**Figure 5:**
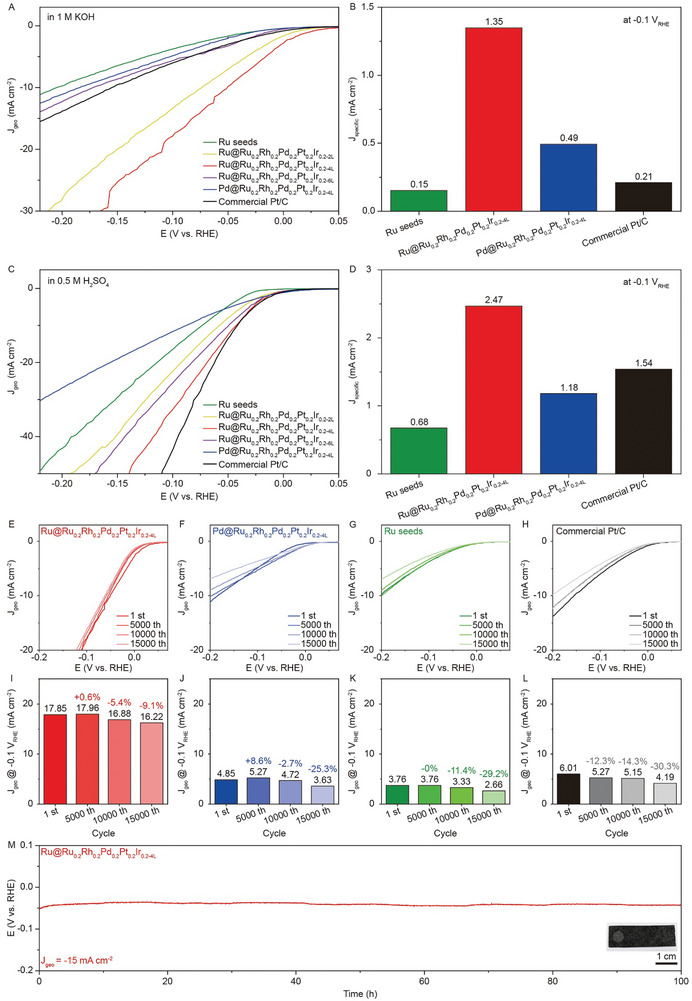
Electrocatalytic HER performances of HCP Ru@Ru_0.2_Rh_0.2_Pd_0.2_Pt_0.2_Ir_0.2‐nL_ and FCC Pd@Ru_0.2_Rh_0.2_Pd_0.2_Pt_0.2_Ir_0.2‐4L_ core‐shell nanocrystals. A,B) HER in 1.0 m KOH solution: A) Polarization curves and B) specific activities at −0.1 V_RHE_. C,D) HER in 0.5 m H_2_SO_4_ solution: C) Polarization curves and D) specific activities at ‐0.1 V_RHE_. E–L) Durability tests of (E, I) HCP Ru@Ru_0.2_Rh_0.2_Pd_0.2_Pt_0.2_Ir_0.2‐4L_, (F, J) FCC Pd@Ru_0.2_Rh_0.2_Pd_0.2_Pt_0.2_Ir_0.2‐4L_, (G, K) Ru seeds, and (H, L) commercial Pt/C in 1.0 m KOH solution. (M) Chronopotentiometry measurement of HCP Ru@Ru_0.2_Rh_0.2_Pd_0.2_Pt_0.2_Ir_0.2‐4L_ dispersed on a carbon paper support (inset) at −15 mA cm^−2^ in 1.0 m KOH solution.

The platinum‐group metals are widely recognized as promising electrocatalysts for both alkaline and acidic water electrolysis, offering an attractive method to generate environmentally friendly hydrogen energy.^[^ ^8^, ^9^, ^10^, ^11^, ^14^, ^15^, ^19^, ^20^, ^21^ ^]^ However, so far, the limited HER activity and durability have hindered their applications, particularly in the context of alkaline water electrolysis. Therefore, we investigate the impact of crystal structure on the catalytic performance of Ru@RuRhPdPtIr core−shell nanocrystals, with the aim of attaining superior catalytic activity and long‐term durability. In the first set of electrochemical measurements, we examine the electrocatalytic HER activities of HCP Ru@Ru_0.2_Rh_0.2_Pd_0.2_Pt_0.2_Ir_0.2‐nL_ and FCC Pd@Ru_0.2_Rh_0.2_Pd_0.2_Pt_0.2_Ir_0.2‐4L_ samples in an alkaline 1.0 m KOH electrolyte, as shown in **Figure** **5**A,B. The corresponding catalysts, including commercial Pt/C and Ru seeds, are used as references. The linear sweep voltammetry (LSV) curves of the tested catalysts show that the HCP Ru@Ru_0.2_Rh_0.2_Pd_0.2_Pt_0.2_Ir_0.2‐4L_ core‐shell nanocrystals exhibit the lowest HER overpotential of 50.8 mV (vs the reversible hydrogen electrode, RHE) at a current density of −10 mA cm^−2^ (where the current is normalized by geometric electrode area of 0.07 cm^2^ (Figure S17, Supporting Information)). To further characterize their intrinsic activities, the values of specific activities (where the current is normalized by the electrochemically active surface area, ECSA (Figure S18, Supporting Information)) at a potential of −0.1 V are compared in Figure 5B. The HCP Ru@Ru_0.2_Rh_0.2_Pd_0.2_Pt_0.2_Ir_0.2‐4L_ core‐shell nanocrystals reach the highest specific activity of 1.35 mA cm^−2^ at −0.1 V, which is 2.8, 6.4, and 9.0 times higher than those of their FCC counterparts (0.49 mA cm^−2^), commercial Pt/C reference (0.21 mA cm^−2^), and Ru seeds (0.15 mA cm^−2^), respectively.

In the second set of electrochemical measurements, we study the electrocatalytic HER activities in an acidic 0.5 m H_2_SO_4_ electrolyte, as shown in Figure 5C,D. Again, the HCP Ru@Ru_0.2_Rh_0.2_Pd_0.2_Pt_0.2_Ir_0.2‐4L_ samples show a much better HER electrocatalytic activity than the FCC Pd@Ru_0.2_Rh_0.2_Pd_0.2_Pt_0.2_Ir_0.2‐4L_ samples. The values of the specific activities at an overpotential of −0.1 V of the HCP Ru@Ru_0.2_Rh_0.2_Pd_0.2_Pt_0.2_Ir_0.2‐4L_ samples is 2.47 mA cm^−2^, which is 2.1, 1.6, and 3.6 times higher than those of their FCC counterparts (1.18 mA cm^−2^), commercial Pt/C reference (1.54 mA cm^−2^), and Ru seeds (0.68 mA cm^−2^), respectively. It should be emphasized that the HCP Ru@Ru_0.2_Rh_0.2_Pd_0.2_Pt_0.2_Ir_0.2‐4L_ nanocatalysts exhibit a more significant improvement in specific activity in the alkaline electrolyte (6.4 times) compared to their performance in the acidic solution (1.6 times) when benchmarked against commercial Pt/C catalysts. The electrochemical impedance spectroscopy (EIS) measurements for HCP Ru@Ru_0.2_Rh_0.2_Pd_0.2_Pt_0.2_Ir_0.2‐4L_ core‐shell nanocrystals and commercial Pt/C catalysts are conducted under various overpotentials of 0, −0.05, −0.1, and −0.15 V, as shown in Figure S19 (Supporting Information). The detailed measurement procedures are provided in the updated supporting information. The Nyquist plots in Figure S19 (Supporting Information) reveal a trend of decreasing resistance with increasing overpotentials. Notably, the Ru@Ru_0.2_Rh_0.2_Pd_0.2_Pt_0.2_Ir_0.2‐4L_ core‐shell nanocrystals (Figure S19A, Supporting Information) display a smaller semicircle across the applied potentials compared to the Pt/C catalyst (Figure S19B, Supporting Information), indicating a lower charge transfer resistance and more efficient electron transfer at the electrode‐electrolyte interface during the HER process.^[^ ^42^, ^43^ ^]^ This aligns with the enhanced catalytic performance observed in these Ru@Ru_0.2_Rh_0.2_Pd_0.2_Pt_0.2_Ir_0.2‐4L_ core‐shell nanocrystals. These results highlight the unconventional HCP HEA nanocatalysts as excellent HER catalysts, particularly in alkaline environments.

Given that the degradation of commercial Pt/C is more pronounced in an alkaline solution than in an acidic electrolyte, we thus focus on the durability tests of our catalysts for alkaline HER. As shown in Figure 5E, there is no significant change in the LSV curves for the HCP Ru@Ru_0.2_Rh_0.2_Pd_0.2_Pt_0.2_Ir_0.2‐4L_ samples after 5000, 10000, and 15000 cycles. In contrast, the FCC Pd@Ru_0.2_Rh_0.2_Pd_0.2_Pt_0.2_Ir_0.2‐4L_, commercial Pt/C, and Ru seeds exhibit a gradual decrease in activity throughout the durability tests (Figure 5F–H). As shown in Figure 5I, after 15000 cycles, the HCP Ru@Ru_0.2_Rh_0.2_Pd_0.2_Pt_0.2_Ir_0.2‐4L_ samples demonstrate only a 9.1% decrease in catalytic activity at a potential of −0.1 V, whereas their FCC counterparts, commercial Pt/C, and Ru seeds experience drops of 25.3%, 30.3%, and 29.2% in activity, respectively (Figure 5J–L). Additionally, the Ru@Ru_0.2_Rh_0.2_Pd_0.2_Pt_0.2_Ir_0.2‐4L_ nanocrystals dispersed on the conductive carbon paper substrate also demonstrate no significant change in overpotential, with a fluctuation of less than 20% at a constant current density of −15 mA cm^−2^ over a 100 h operating period (Figure 5M). By the end of the durability test, the final overpotential decreases slightly from an initial value of ≈−52 to ≈−43 mV. This modest improvement in catalytic activity over the 100 h period is likely due to the gradual removal of surface oxides during the HER. Based on previous studies,^[^ ^44^, ^45^, ^46^, ^47^ ^]^ a 100 h chronoamperometric test at a high current density of −15 mA cm^−2^ is deemed sufficient to assess the durability of catalysts under alkaline HER conditions. The TEM, HAADF‐STEM images, and EDS mappings indicate that the structure and homogeneous distribution of the elements in most HCP Ru@Ru_0.2_Rh_0.2_Pd_0.2_Pt_0.2_Ir_0.2‐4L_ samples are maintained after the durability tests (Figure S20, Supporting Information). Additionally, the ICP‐OES results reveal no detectable element dissolution. These results suggest that the HCP Ru@Ru_0.2_Rh_0.2_Pd_0.2_Pt_0.2_Ir_0.2‐4L_ samples have superior durability during the alkaline HER. Due to the small amount of catalyst used, with only 0.2 mg of Ru@Ru_0.2_Rh_0.2_Pd_0.2_Pt_0.2_Ir_0.2‐4L_ with only 4 atomic layers of HEA shells dispersed on the Vulcan XC72R carbon and mixed with the Nafion solution to form catalyst ink, which is then drop‐cast onto a glassy carbon electrode for HER electrocatalytic measurements, it is challenging to recover a sufficient amount for XPS analysis. However, since the HER is conducted under negative potentials, oxidation of the samples is unlikely, and the metals are expected to remain predominantly in their metallic states. This inference is supported by the durability results of HCP Ru@Ru_0.2_Rh_0.2_Pd_0.2_Pt_0.2_Ir_0.2‐4L_ samples. As referenced in recent literature,^[^ ^48^, ^49^ ^]^ HEA‐based materials exhibit enhanced stability due to the absence of preferential initiation sites for degradation or phase separation. The random distribution of elements in HEA‐based materials minimizes the formation of grain boundaries or corrosion‐prone sites, further supported by the uniform atomic distribution observed in EDS mappings and synchrotron XAS analysis in this study. Also, our findings show that the HCP Ru@Ru_0.2_Rh_0.2_Pd_0.2_Pt_0.2_Ir_0.2‐4L_ nanocrystals demonstrate superior durability compared to their FCC counterparts. This aligns with other studies on HEA‐based materials, which have shown that their unique atomic arrangements delay or prevent electrochemical degradation.^[^ ^49^ ^]^

### 2.4 Electrocatalytic HER Mechanism and Synergistic Effect Unveiled by Electrochemical Analysis and Operando XAS Analysis

The two‐electron transfer alkaline HER consists of three elementary steps, in which active catalysts (M) are required to reduce the energy barriers in each step:

(1) $${M+H_{2}O+e}^{-}\to {\;M-H_{\mathrm{ad}}+\mathrm{OH}}^{-}\;\left(\mathrm{Volmer}\;\mathrm{step}\right)$$

(2) $${M-H_{\mathrm{ad}}+H_{2}O+e}^{-}\;\to {\;M+H_{2}+\mathrm{OH}}^{-}\;\left(\mathrm{Heyrovsky}\;\mathrm{step}\right)$$

(3) $$M-H_{\mathrm{ad}}+M-H_{\mathrm{ad}}\to \;2\;M+H_{2}\;\left(\mathrm{Tafel}\;\mathrm{step}\right)$$

The generally accepted pathways are associated with the water dissociation and adsorption/desorption of a hydrogen intermediate (H*) on the catalysts through either the Volmer‐Heyrovsky or the Volmer‐Tafel mechanism.^[^ ^50^, ^51^, ^52^, ^53^, ^54^, ^55^ ^]^ The water dissociation in both Volmer and Heyrovsky steps requires considerable energy to break the HO‐H bond, contributing to the slower reaction rate observed in alkaline HER. Importantly, recent simulations also suggest the binding strength of hydroxyl (OH*) also plays an important role in hydrogen evolution reaction kinetics. When catalysts exhibit a weak binding affinity for OH*, the rate‐limiting step is often the dissociation of water. Conversely, as the strength of OH* binding increases, the adsorption of OH* occurs on the catalysts, leading to a slower OH* desorption rate. Based on their 3D volcano plot, the rate of hydrogen evolution is further determined by the strength of binding between hydrogen/hydroxyl and the catalyst surface.^[^ ^56^ ^]^ It is expected that catalysts with a moderate binding strength of hydrogen/hydroxyl exhibit high alkaline HER activity.

**Figure 6:**
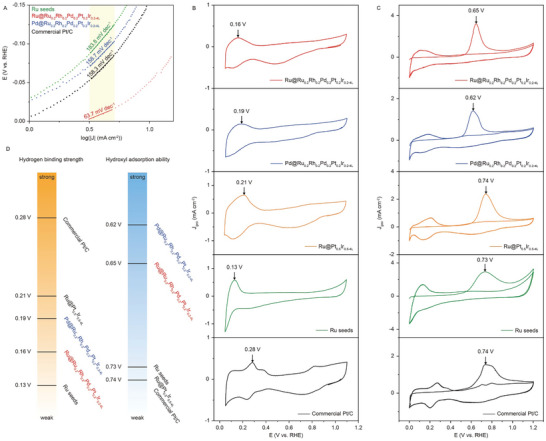
Electrochemical characterization for revealing the catalytic mechanism of alkaline HER. A) Tafel plots of HCP Ru@Ru_0.2_Rh_0.2_Pd_0.2_Pt_0.2_Ir_0.2‐4L_, FCC Pd@Ru_0.2_Rh_0.2_Pd_0.2_Pt_0.2_Ir_0.2‐4L_, Ru seeds and commercial Pt/C obtained from corresponding polarization curves. B) CV curves and C) CO stripping curves of HCP Ru@Ru_0.2_Rh_0.2_Pd_0.2_Pt_0.2_Ir_0.2‐4L_, FCC Pd@Ru_0.2_Rh_0.2_Pd_0.2_Pt_0.2_Ir_0.2‐4L_, HCP Ru@Pt_0.5_Ir_0.5‐4L_, Ru seeds and commercial Pt/C. D) A summary of hydrogen binding strength and hydroxyl adsorption ability according to the potentials of HUPD desorption peaks and CO oxidation peaks.

In general, the Tafel slope serves as a good indicator of reaction kinetics and the rate‐determining step (RDS) in electrocatalytic reactions.^[^ ^57^ ^]^ Varying values of the Tafel slope typically correspond to different reaction mechanisms and RDS. As shown in **Figure** **6**A, the HCP Ru@Ru_0.2_Rh_0.2_Pd_0.2_Pt_0.2_Ir_0.2‐4L_ catalysts exhibit a Tafel slope value of 63.7 mV dec^−1^, suggesting that the HER follows the Volmer‐Heyrovsky pathway with the Heyrovsky step being the RDS.^[^ ^58^, ^59^ ^]^ This RDS involves the cleaving of the HO‐H bond to react with the adsorbed hydrogen on the catalysts, leading to the formation of a hydrogen molecule and a hydroxyl species. However, as the layer thickness increases to six, either the Volmer‐Tafel or Volmer‐Heyrovsky pathway becomes dominant, with the Volmer step as the RDS (Figure S21, Supporting Information). According to the Tafel‐slope findings in this study, the alkaline HER activity is governed by the binding strength of both intermediates H* and OH* involved in the rate‐determining Heyrovsky step. To gain deeper insights into the catalytic mechanism, we utilize electrochemical desorption curves for hydrogen underpotential deposition (HUPD; Figure 6B) and CO‐stripping curves (Figure 6C) for both the HCP Ru@Ru_0.2_Rh_0.2_Pd_0.2_Pt_0.2_Ir_0.2‐4L_ and FCC Pd@Ru_0.2_Rh_0.2_Pd_0.2_Pt_0.2_Ir_0.2‐4L_ catalysts. They are convincing electrochemical tools for examining the binding affinity of H* and OH* on the catalysts, respectively.^[^ ^59^, ^60^ ^]^ Additionally, the corresponding catalysts, including commercial Pt/C, Ru seeds, and Ru@Pt_0.5_Ir_0.5‐4L_ samples, are used as references. In the cyclic voltammetry (CV) experiments for the HUPD, stronger hydrogen binding can be identified by observing the shift of HUPD peaks toward higher potentials. As shown in Figure 6B,D, the trend of HUPD peaks for the catalysts is as follows: commercial Pt/C (0.28 V) > Ru@Pt_0.5_Ir_0.5‐4L_ (0.21 V) > FCC Pd@Ru_0.2_Rh_0.2_Pd_0.2_Pt_0.2_Ir_0.2‐4L_ (0.19 V) > HCP Ru@Ru_0.2_Rh_0.2_Pd_0.2_Pt_0.2_Ir_0.2‐4L_ (0.16 V) > Ru seeds (0.13 V). These results suggest that the binding strength of H_ad_ on HCP HEA structures falls within a moderate range of strength when compared to the FCC HEA and the other mono‐ or bi‐metallic catalysts. In addition, we performed CO stripping experiments to evaluate the binding affinity of OH* since OH* can promote the removal of surface‐adsorbed CO* intermediates into CO_2_. Typically, lower potentials of CO‐stripping peaks indicate higher abilities for OH adsorption. As shown in Figure 6C,D, the trend of CO‐stripping peaks for the catalysts can be summarized as follows: FCC Pd@Ru_0.2_Rh_0.2_Pd_0.2_Pt_0.2_Ir_0.2‐4L_ (0.62 V) < HCP Ru@Ru_0.2_Rh_0.2_Pd_0.2_Pt_0.2_Ir_0.2‐4L_ (0.65 V) < Ru seeds (0.73 V) < Ru@Pt_0.5_Ir_0.5‐4L_ (0.74 V) ≈ commercial Pt/C (0.74 V). We compared the CO‐stripping peaks of different catalysts by identifying the peak potential at which the maximum current density occurs. The CO‐stripping peak for FCC HEA displays a shoulder, likely attributed to the presence of different facets on the catalyst surface, a feature also observed in commercial Pt/C.^[^ ^61^ ^]^ Again, the HCP Ru@Ru_0.2_Rh_0.2_Pd_0.2_Pt_0.2_Ir_0.2‐4L_ sample exhibits a moderate binding strength compared to these catalysts. The oxidation of adsorbed CO is easier on the FCC Pd@Ru_0.2_Rh_0.2_Pd_0.2_Pt_0.2_Ir_0.2‐4L_ compared to the HCP Ru@Ru_0.2_Rh_0.2_Pd_0.2_Pt_0.2_Ir_0.2‐4L_, due to the stronger adsorption of OH_ad_ on the FCC HEA surface. The Pd@Ru_0.2_Rh_0.2_Pd_0.2_Pt_0.2_Ir_0.2‐4L_ sample, possessing the strongest adsorption affinity for OH* among these catalysts, may result in a slower OH* desorption rate during water dissociation. Conversely, for samples like Ru seeds, Ru@Pt_0.5_Ir_0.5‐4L_, and commercial Pt/C, which exhibit a much weaker binding affinity for OH*, the rate of water dissociation on the surface could be limited.

**Figure 7:**
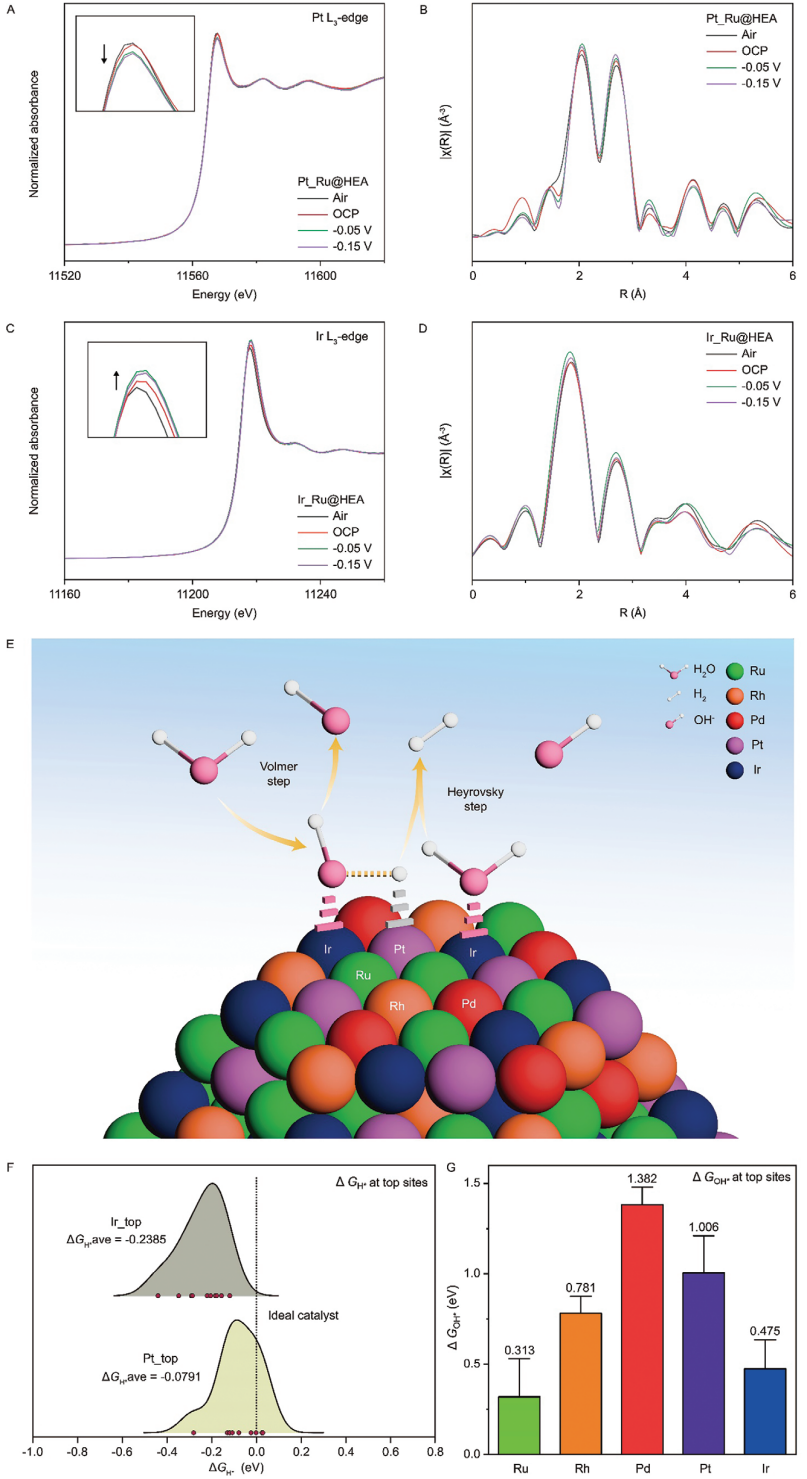
*Operando* synchrotron XAS measurements of Ru@Ru_0.2_Rh_0.2_Pd_0.2_Pt_0.2_Ir_0.2‐4L_ under alkaline HER and schematic of catalytic mechanism. A,B) *Operando* XANES and FT‐EXAFS of Pt L_3_‐edge under the applied potential from OCP, −0.05 to −0.15 V. C,D) *Operando* XANES and FT‐EXAFS of Ir L_3_‐edge under the applied potential from OCP, −0.05 to −0.15 V. E) Schematic of the alkaline HER catalytic mechanism: Pt sites adsorb H*, while Ir sites facilitate water dissociation and the adsorption and desorption of OH*. F) Adsorption strength of H* at the top sites of Pt and Ir. G) Adsorption strength of OH* at the top sites of elements on the HCP RuRhPdPtIr surface.

Furthermore, *operando* XAS measurements are utilized to elucidate the synergistic electronic interactions among the mixed elements and identify the actual active sites with possible intermediates involved in the alkaline HER mechanism for the representative HCP Ru@Ru_0.2_Rh_0.2_Pd_0.2_Pt_0.2_Ir_0.2‐4L_ sample, as shown in **Figure** **7**.^[^ ^8^, ^21^, ^62^, ^63^, ^64^ ^]^ The measurements are performed for Ru, Rh, Pd, Pt, and Ir elements under both open circuit potential (OCP) and applied potentials from −0.05 to −0.15 V through XANES. Interestingly, when the applied potential switch from OCP to −0.15 V, where the HER launched, there is a decrease in the white line intensity of the Pt L_3_‐edge (Figure 7A), accompanied by an increase in the white line intensity of the Ir L_3_‐edge (Figure 7C). This phenomenon indicates a significant transfer of electrons from Ir sites to Pt sites and/or the adsorption of reaction intermediates on both Pt and Ir sites during the alkaline HER. This results in electron gain at Pt sites and electron donation at Ir sites. During the Volmer step, H* preferentially adsorbs on Pt active sites of HCP HEA, while Ir sites actively participate in water dissociation, followed by the adsorption and desorption of OH*. Subsequently, another water molecule adsorbed on Ir sites reacts with the adsorbed H* to generate H_2_ in the Heyrovsky step (Figure 7E). This complementary interaction between Pt and Ir should likely occur between adjacent Pt and Ir atoms (Table 1), as isolated Pt and Ir atoms would be too far apart to effectively enhance the overall catalytic performance. The proximity of these Pt and Ir atoms allows for efficient synergy, demonstrating the crucial roles of these active sites in optimizing the HER process.

It is noteworthy that the absorption edges of Ru, Rh, and Pd K‐edges remained largely unchanged during the HER, suggesting that these three elements on the HCP HEA surface are not direct active sites (Figure S22, Supporting Information). Importantly, our control experiments showed that the specific activity of bimetallic Ru@Pt_0.5_Ir_0.5‐4L_ (0.60 mA cm^−2^) is much lower than that of the Ru@Ru_0.2_Rh_0.2_Pd_0.2_Pt_0.2_Ir_0.2‐4L_ (1.35 mA cm^−2^) (Figure S23, Supporting Information). These experiments indicate that the presence of non‐active Ru, Rh, and Pd elements mixed with active Pt and Ir elements could enhance the configurational entropy and finely tune the electronic structure of the HEA surface through synergistic effects among the elements, which are beneficial for the catalytic performance. These synergistic effects can also be confirmed through the HUPD and CO‐stripping experiments (Figure 6B–D), demonstrating that the binding strength of H* and OH* can be weakened and strengthened to a moderate level, respectively, by mixing non‐active Ru, Rh, and Pd atoms with active Pt and Ir atoms. Furthermore, the corresponding FT‐EXAFS spectra of Pt and Ir in the Ru@Ru_0.2_Rh_0.2_Pd_0.2_Pt_0.2_Ir_0.2‐4L_ display negligible changes as the applied potential varies during the alkaline HER (Figure 7B,D). This observation also suggests their durability. Together, the synergistic effects among the mixed elements in the HCP HEA structure could lead to moderate binding strengths of H* and OH* on the active Pt and Ir sites, respectively, which could contribute to improved catalytic performance in alkaline HER. In this study, the composition, ratios, and atomic layers of the Ru_0.2_Rh_0.2_Pd_0.2_Pt_0.2_Ir_0.2‐4L_ HEA on the HCP Ru and FCC Pd seeds are controlled to be similar, with the primary difference being the HCP versus FCC structural variations and the different seed materials used. Despite the similar electronegativities of Ru and Pd (both 2.20), which suggest comparable electronic interactions with the seeds, differences in lattice mismatch and thus strain effects between the seeds and HEA shells likely contribute to the observed variations in electronic interactions (Tables S4 and S5, Supporting Information).^[^ ^65^, ^66^, ^67^ ^]^ Therefore, the distinct electronic interactions in HCP and FCC HEA structures are likely due to differences in atomic stacking sequences, surface atomic arrangements, and strain effects within the HEA surfaces. It should be emphasized that the synergistic effect observed in multi‐component high‐entropy materials is recognized as a complex and intertwined mechanism that is crucial for achieving exceptional catalytic performance.^[^ ^4^, ^8^, ^9^, ^10^, ^11^ ^]^ The nature of understanding the intricate workings of multi‐component synergy within HEA is still elusive. Further investigations using advanced techniques, such as high‐throughput DFT computations,^[^ ^68^ ^]^ could significantly enhance our understanding of the synergistic effects of HEA catalysts.^[^ ^2^, ^69^, ^70^, ^71^ ^]^

We performed DFT calculations to study the adsorption free energies of H^*^ (ΔG_H*_) and OH^*^ (ΔG_OH*_) on the HCP RuRhPdPtIr surface.^[^ ^8^, ^72^ ^]^ For the ΔG_H*_, we sample more than 150 possible atomic configurations of the H‐adsorbed PdPtRhIrRu surface including top, hollow, and bridge sites, resulting in ≈80 unique H‐adsorption configurations after geometry optimization. In the case of top sites, we find that the majority of hydrogen migrates to the top sites of Pt and Ir compared to those of Ru, Rh, and Pd. This suggests that Pt and Ir provide the most favorable H adsorption sites on the HCP RuRhPdPtIr surface. Additionally, the adsorption free energy of Pt (−0.0791 eV) is closer to the ideal value (0 eV) than Ir (−0.2385 eV), as shown in Figure 7F. This comparison indicates that the Pt top site is an ideal active site for HER, with adsorption strength that is neither too strong nor too weak, aligning with the Sabatier principle. Ir, by contrast, shows stronger hydrogen adsorption, which may not be as optimal as Pt. This trend is also observed in the adsorption of hydrogen on the hollow and bridge sites (Figure S24, Supporting Information). This consistency across different adsorption sites further highlights the role of Pt in providing optimal H‐adsorption sites on the HCP RuRhPdPtIr surface, consistent with *operando* XAS measurements (Figure 7A).

For the ΔG_OH*_, we positioned OH molecules on the top sites of each metal atom on the HCP RuRhPdPtIr surface. Ideally, the ΔG_OH*_ should be moderate, neither too strong nor too weak, to facilitate efficient catalysis. A moderately enhanced OH^*^ adsorption strength can create potential active sites on the HCP RuRhPdPtIr surface. However, to optimize the conversion from OH_ad_ to OH^−^, the desorption ability also plays a key role, as it is closely related to the surface charge properties and is crucial for catalytic performance. Interestingly, our calculations reveal that ΔG_OH*_ values at the top sites of Ir and Ru exhibit stronger adsorption compared to those of Rh, Pd, and Pt (Figure 7G). Specifically, the calculated ΔG_OH*_ for Ru (0.313 eV) and Ir (0.475 eV) at top sites suggest that these elements are the most oxophilic sites.^[^ ^72^ ^]^ Nevertheless, significantly stronger OH adsorption of Ru (0.313 eV) may impede OH desorption, potentially slowing subsequent HER steps. This strong OH binding is also observed at the top, hollow, and bridge sites where Ir or Ru are present (Figure S25, Supporting Information). This inference is further supported by the unchanging *operando* XAS signals for Ru under different applied potentials during alkaline HER, suggesting that its adsorption strength may be too strong, thus preventing efficient OH desorption (Figure S22A, Supporting Information). Therefore, these DFT results suggest that Ir offers a more balanced OH adsorption behavior compared to the other elements on the HCP RuRhPdPtIr surface. They align with *operando* XAS measurements obtained in this study, where Ir is identified as an electron‐donating site (Figure 7C), enabling efficient conversion of OH_ad_ to OH⁻ (OH_ad_ + e⁻ → OH⁻),^[^ ^73^ ^]^ thereby accelerating the Volmer step. Our experimental findings may open new avenues for the rational design of unconventional HEA structures with strong atomic‐scale interactions for electrocatalytic reactions involving multiple intermediates.

## 3 Conclusion

In this study, we demonstrate unconventional HCP RuRhPdPtIr HEA atomic layers using a method involving the dropwise addition of metal precursor mixtures in the presence of HCP Ru seeds, facilitating layer‐by‐layer epitaxial growth. Their HCP structures can be obtained within ≈6 atomic layers but change to the thermodynamically stable FCC structure as the thickness increases further. The XAS fitting results of HCP RuRhPdPtIr HEA reveal that the five constituent elements can bond with each other and lead to unique electronic interactions, distinguishing them from the traditional FCC RuRhPdPtIr HEA. Additionally, HCP RuRhPdPtIr HEA atomic layers are stable even at temperatures up to 500 °C, confirmed by HRPXRD analysis with in situ heating. Most importantly, HCP RuRhPdPtIr HEA layers exhibit notable improvements in both electrocatalytic activity and durability for the HER in an alkaline environment, as compared to their FCC RuRhPdPtIr counterparts. Interestingly, they exhibit a more significant improvement in activity in the alkaline electrolyte (6.4 times) compared to their performance in the acidic solution (1.6 times) when benchmarked against commercial Pt/C catalysts. Furthermore, *operando* XAS analyses reveal electron enrichment at Pt sites and electron withdrawal at Ir sites on the HCP HEA surfaces during the alkaline HER, indicating preferential adsorption of H* on Pt sites and OH* on Ir sites. Additionally, the electrochemical HUPD desorption analyses and CO‐stripping experiments also indicate that the binding strengths of H* and OH* intermediates on the HEA can be weakened and strengthened to a moderate level, respectively, by incorporating non‐active Ru, Rh, and Pd atoms with active Pt and Ir atoms. These results highlight the importance of engineering unconventional HEA structures and surfaces with appropriate binding strengths for intermediate species when designing high‐performance alkaline HER catalysts. This approach can potentially be extended to other multi‐component catalysts and catalytic reactions involving multiple intermediates.

## Conflict of Interest

The authors declare no conflict of interest.

## Supporting information



Supporting Information

Supplemental Video 1

## Data Availability

The data that support the findings of this study are available from the corresponding author upon reasonable request.
