# Supplementary material for: Unconventional Hexagonal Close‐Packed High‐Entropy Alloy Surfaces Synergistically Accelerate Alkaline Hydrogen Evolution
Source: Adv Sci (Weinh). 2024 Nov 8;12(1):2409023. doi: 10.1002/advs.202409023 (PMC11714166; doi:10.1002/advs.202409023)
Supplement: Supplementary file 1 — Supporting Information [file ADVS-12-2409023-s001.docx]

Supporting information

**Unconventional Hexagonal Close-Packed High-Entropy Alloy Surfaces Synergistically Accelerate Alkaline Hydrogen Evolution**

*Ting-Hsin Hu^+^, Cheng-Yu Wu^+^, Zong Ying He, Yi Chen, Liang-Ching Hsu, Chih-Wen Pao,* *Jui-Tai Lin, Chun‐Wei Chang, Shang-Cheng Lin,* *Rachel Osmundsen, Lee Casalena,* *Kun Han Lin, Shan Zhou, and* *Tung-Han Yang**

**This Supporting Information file includes:**

**Materials and Methods, Figures S1 to S25, Tables S1 to S5,** **Video S1, and References**

**Materials and Methods**

**Chemicals.** Ruthenium acetylacetonate (Ru(acac)_3_), rhodium acetylacetonate (Rh(acac)_3_), palladium acetylacetonate (Pd(acac)_2_), platinum acetylacetonate (Pt(acac)_2_), hydrogen hexachloroiridate hydrate (H_2_IrCl_6_·xH_2_O), iridium acetylacetonate (Ir(acac)_3_), iron acetylacetonate (Fe(acac)_3_), nickel acetylacetonate (Ni(acac)_2_), silver acetylacetonate (Ag(acac)), sodium tetrachloropalladate (Na_2_PdCl_4_), L-ascorbic acid (AA), polyvinylpyrrolidone (PVP, M_W_ ≈ 55,000), and potassium bromide (KBr) are all purchased from Sigma-Aldrich and utilized after received. Ethylene glycol (EG) and triethylene glycol (TEG) are purchased from J. T. Baker. Deionized (DI) water (18.2 MΩ·cm) is employed for all experiments.

**Synthesis of 6.7-nm Ru seeds.** To synthesize the 6.7-nm Ru seeds, a standardized procedure is followed.^[1]^ Initially, 5 mL of EG solution containing PVP (50 mg) and Ru(acac)_3_ (7.5 mg) is introduced into a 23-mL vial. Subsequently, the mixture is heated to 160 °C and reacts for 3 h under magnetic stirring, forming the 3-nm Ru seeds. Following the reaction, the solution is promptly cooled in an ice bath to terminate the reaction process. For the further synthesis of 7-nm Ru seeds, the preformed 3-nm Ru seeds are heated again to 160 °C. Consequently, 5 mL of EG containing 150 mg of Ru(acac)_3_ and 50 mg PVP is injected into the solution at a rate of 2 mL/h. After the injection, the reaction mixture is maintained at 160 °C for an additional 0.5 h under magnetic stirring and then cooled to room temperature. The Ru seeds are dispersed in EG solvent after a cleaning procedure involving centrifugation and washing with acetone once and DI water three times.

**Synthesis of 7.5-nm Pd seeds.** To synthesize the 7.5-nm Pd seeds, a standardized procedure is followed.^[2]^ An 8-mL aqueous solution containing AA (60 mg), and PVP (105 mg) is added to a 23-mL vial. This solution is then pre-heated at 50 °C for 1 min. Following the pre-heating step, a single 3-mL injection of an aqueous Na_2_PdCl_4_ solution with a concentration of 19 mg/mL is administrated into the vial. The reaction mixture is sealed and maintained at 50 °C for 3 h under magnetic stirring and then cooled to room temperature. The resulting Pd seeds are subsequently isolated through a cleaning procedure that includes centrifugation and washing with DI water three times and then dispersed in EG solvent.

**Synthesis of HCP Ru@HEA and FCC Pd@HEA core-shell nanocrystals using epitaxial growth.** The HCP and FCC RuRhPdPtIr HEA atomic layers are synthesized on Ru and Pd seeds through epitaxial growth, respectively. Initially, 1 mL of an EG solution containing Ru/Pd seeds is combined with 3 mL EG containing PVP (100 mg), KBr (60 mg), and AA (20 mg), in a 23-mL vial. This mixture is pre-heated at 110 °C for 10 min under magnetic stirring, followed by an increase in temperature to 160 °C for 20 min. A 14-mL EG precursor solution containing Ru(acac)_3_ (0.702 mg), Rh(acac)_3_ (1.058 mg), Pd(acac)_2_ (0.268 mg), Pt(acac)_2_ (0.693 mg), and H_2_IrCl_6_·xH_2_O (0.813 mg) is then prepared. Employing a syringe pump, this precursor solution is added dropwise to the pre-heated mixture containing Ru/Pd seeds at a constant injection rate of 0.8 mL/h. Following the addition of the precursor solution, the reaction mixture is maintained at 160 °C for an additional 1.5 h. Subsequently, the resulting Ru@RuRhPdPtIr or Pd@RuRhPdPtIr core-shell nanocrystals are collected through centrifugation, washed once with acetone and two times with DI water, and then redispersed in DI water. For the synthesis of octonary RuRhPdPtIrAgFeNi atomic layers on the Ru seeds, the solvent is changed to TEG instead of EG. The TEG solution containing Ru seeds, PVP, KBr and AA is pre-heated at 110 °C for 10 min under magnetic stirring, followed by an increase in temperature to 240 °C for 20 min. For the additional metal precursors employed, Fe(acac)_3_, Ni(acac)_2_, and Ag(acac) corresponded to the elements Fe, Ni, and Ag, respectively. A precursor solution containing eight metal precursors is added dropwise to the pre-heated mixture at the injection rate of 0.6 mL/h, and the reaction mixture is maintained at 240 °C for an additional 1.5 h. The Ru@RuRhPdPtIrAgFeNi core-shell nanocrystals are collected through centrifugation, washed once with acetone and two times with DI water, and then redispersed in DI water.

**Electrocatalytic measurements for hydrogen evolution reaction (HER).** In this study, HER electrochemical measurements are performed using a Metrohm Autolab PGSTAT204 Nova electrochemical workstation. A glassy carbon electrode is used as the working electrode for rotating electrodes, with platinum as the counter electrode. For the reference electrode, Hg/HgO and Ag/AgCl electrodes are used in 1.0 M KOH and 0.5 M H_2_SO_4_ electrolytes, respectively. To formulate the catalyst ink, 1 mg of catalysts on Vulcan XC72R carbon support (containing 20 wt%) are mixed with 0.5 mL of isopropanol, 0.5 mL DI water, and 0.02 mL of a 5% Nafion solution. This mixture is homogenized in an ice-cooled ultrasonic bath. Subsequently, 3.6 μL of this catalyst ink is applied to a glassy carbon electrode with a surface area of 0.07 cm^2^ and allowed to dry in an isopropanol-saturated atmosphere before testing.

Electrochemical characterization for HER investigations is carried out in 1.0 M KOH and 0.5 M H_2_SO_4_ solution, saturated with nitrogen gas. In this study, 150 cycles of cyclic voltammetry (CV) are conducted at a high scan rate of 500 mV/s across a potential range of 0.05 to 1.1 V_RHE_ (reversible hydrogen electrode, RHE) to stabilize the catalysts, ensuring that the electrode surfaces are properly conditioned and free of any surface contaminants. After this typical pre-activation step, linear sweep voltammetry (LSV) is performed, scanning from 0.1 to -0.25 V_RHE_ at 1,600 rpm and 1 mV/s to evaluate the HER activity. Durability testing of the catalysts is performed by cycling 15,000 times at a scan rate of 100 mV/s within the same potential range as the LSV test. The chronopotentiometry measurement is performed under 15 mA cm^-2^ for 100 h, while the metal catalysts (0.3 mg) are dispersed on the carbon paper substrate with an approximate surface area of 0.22 cm^2^ as the working electrode.

In the evaluation of hydrogen adsorption, CV is conducted at a scan rate of 50 mV/s in the range of 0.05 to 1.1 V_RHE_. The peak observed in the CV curve from 0.05 to 0.4 V_RHE_ is analyzed, and the potential of the peak (E_peak_) served as an indicator of hydrogen adsorption strength for each sample according to the equation $\Delta H=-FE_{peak}$.^[3]^

To assess the OH adsorption strength of the catalyst, CO stripping experiments are conducted. Initially, the 1.0 M KOH electrolyte is saturated with CO to ensure complete coverage. A constant voltage is performed at a potential of 0.1 V_RHE_ for 10 min to facilitate CO adsorption onto the catalyst surface. Subsequently, nitrogen gas is purged for another 20 min to expel dissolved CO in the electrolyte. The CV is then carried out for two successive cycles at a scan rate of 50 mV/s in the range of 0 to 1.2 V_RHE_ under the nitrogen purge. The first CV cycle is performed to remove CO from the surface of the catalysts, while the second cycle confirms the absence of CO adsorption.

To evaluate the electrochemical performance of the catalyst in alkaline HER, we perform electrochemical impedance spectroscopy (EIS) measurements for representative HCP Ru@Ru_0.2_Rh_0.2_Pd_0.2_Pt_0.2_Ir_0.2-4L_ core-shell nanocrystals and commercial Pt/C catalysts under various overpotentials of 0, -0.05, -0.1, and -0.15 V vs. RHE in 1 M KOH. At each applied voltage, a small sinusoidal AC voltage of 10 mV is applied to the working electrode over a range of frequencies, typically from 1000 Hz to 0.1 Hz. The corresponding current response is recorded to calculate the impedance of the system. The resulting data are plotted in a Nyquist plot, with the real impedance (Z') plotted along the x-axis and the imaginary impedance (Z'') along the y-axis, allowing for a detailed comparison of the catalysts' charge transfer resistance and electrochemical behavior.

**Evaluation of electrochemical surface area (ECSA).** The electrochemical surface area is measured using the double-layer capacitance (C_dl_). In the measurement of double-layer capacitance, CV with scan rates varying from 25 to 500 mV/s in a 0.5 M N_2_-saturated H_2_SO_4_ electrolyte is utilized. A potential window of 0.1 V, centered at the open circuit potential (OCP), is chosen for the non-faradaic region. To determine the double-layer capacitance, the cathodic and anodic currents are each divided by their corresponding scan rates to compute the slope.^[4]^ The ECSA is then derived using the following formula:

$ECSA= \frac{C_{dl}}{C_{S}}$ (1)

where C_S_ represents a predetermined specific capacitance value.

**X-ray absorption spectroscopy (XAS).** XAS spectra are collected using a customized three-electrode setup in a fluorescence mode. This setup includes a Teflon chamber equipped with a window sealed with Kapton tape. The XAS measurement conditions replicated those utilized for the alkaline HER electrocatalytic tests previously described. During XAS data acquisition, X-rays are transmitted through the Kapton tape, and detection is carried out using total fluorescence yield mode. These measurements are conducted with Lytle detectors located at the TLS-01C1 and TPS-44A beamlines of the National Synchrotron Radiation Research Center (NSRRC, Hsinchu, Taiwan). The obtained data are processed using Athena software to explore the X-ray absorption near-edge structure (XANES) at the K-edge of Ru, Rh, Pd and the L_3_-edge of Pt, Ir. Additionally, Fourier transform (FT) extended X-ray absorption fine structure (EXAFS) analysis is applied, utilizing Fourier transformations on k^2^-weighted EXAFS oscillations to investigate the local coordination environments of the elements of interest, with data fitting conducted using Artemis software.

***In situ*** **synchrotron high-resolution powder X-ray diffraction (HRPXRD) and synchrotron wide angle X-ray scattering (WAXS).** The as-prepared Ru@Ru_0.2_Rh_0.2_Pd_0.2_Pt_0.2_Ir_0.2-4L_ core-shell nanocrystals and Ru seeds are subjected to evaluation using *in situ* HRPXRD at the TPS-19A beamline at the National Synchrotron Radiation Research Center (NSRRC, Hsinchu, Taiwan). The powdered sample is loaded within a capillary of 0.3 mm diameter and is maintained under vacuum conditions to prevent potential oxidation at elevated temperatures. The Ru@Ru_0.2_Rh_0.2_Pd_0.2_Pt_0.2_Ir_0.2-4L_ core-shell nanocrystals and Ru seeds are then heated at a rate of 30 °C per minute under vacuum. The samples are maintained at various temperatures, including room temperature, 100, 200, 300, 400, and 500 °C, to monitor key crystallization signals within the nanocrystals during the thermal annealing process. The WAXS analysis of Ru@Ru_0.2_Rh_0.2_Pd_0.2_Pt_0.2_Ir_0.2-island growth_ core-shell nanocrystals is conducted at the TLS-23A1 beamline of the National Synchrotron Radiation Research Center (NSRRC, Hsinchu, Taiwan).

**Characterizations.** Elemental composition is determined via inductively coupled plasma optical emission spectroscopy (ICP-OES) (Thermo SCIENTIFIC, iCAP 7200 Duo). Sample morphologies are examined using transmission electron microscopy (TEM) and high-angle annular dark-field scanning electron microscopy (HAADF-STEM). For compositional analysis, energy-dispersive spectroscopy (EDS) mapping is conducted using a spherical-aberration corrected field emission TEM (JEOL, JEM-ARM200FTH) at an operated voltage of 200 kV. Additionally, EDS mapping and 3D tomography of Ru@Ru_0.2_Rh_0.2_Pd_0.2_Pt_0.2_Ir_0.2-4L_ (Figure 1G, H and Video S1) are performed using Thermo Scientific Talos F200X STEM scanning transmission electron microscope at the Hillsboro NanoPort. Crystallography analysis is performed using X-ray diffraction (XRD) with an X-ray diffractometer (Bruker, D8). Surface chemistry properties are investigated using X-ray photoelectron spectroscopy (XPS) on a ULVAC-PHI Quantera II high-resolution spectrometer (ULVAC-PHI, PHI Quantera II).


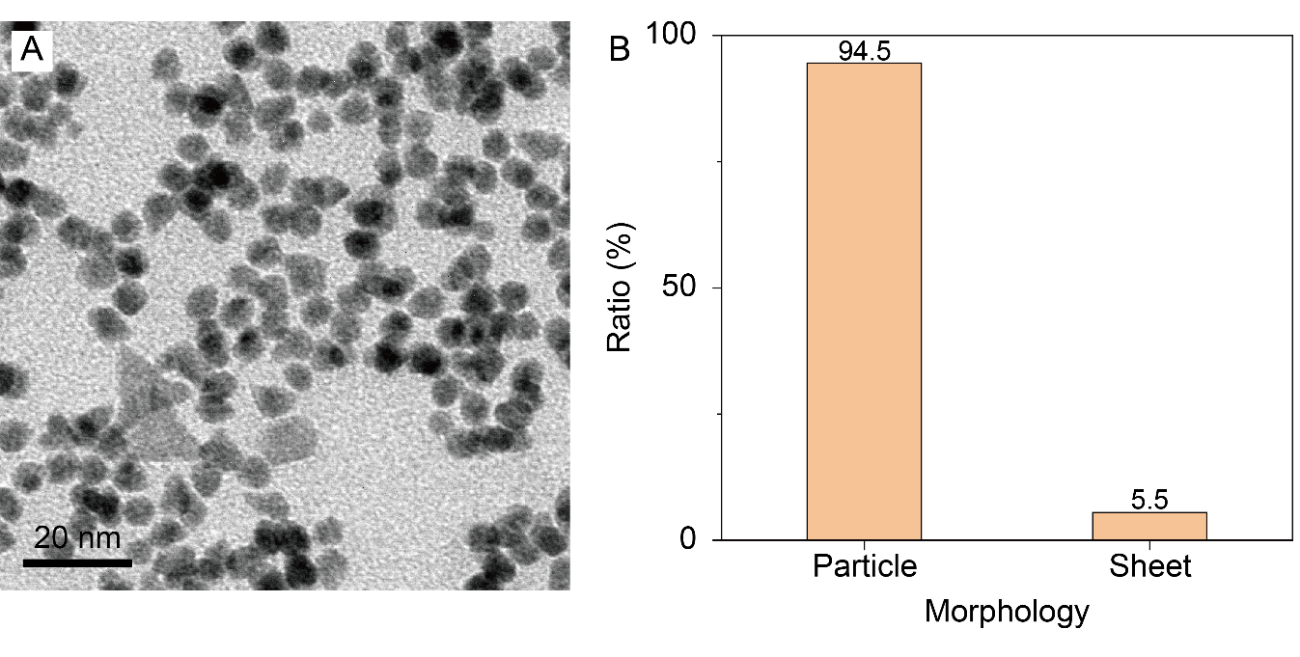


**Figure S1.** (A) TEM image and (B) the morphological statistics of Ru seeds. 94.5% of the Ru seeds exhibit a spherical-like morphology, while 5.5% display a plate-like morphology. The average size of the spherical-like nanocrystals is approximately 6.7 nm. The size of nanocrystals is measured using the ImageJ software.


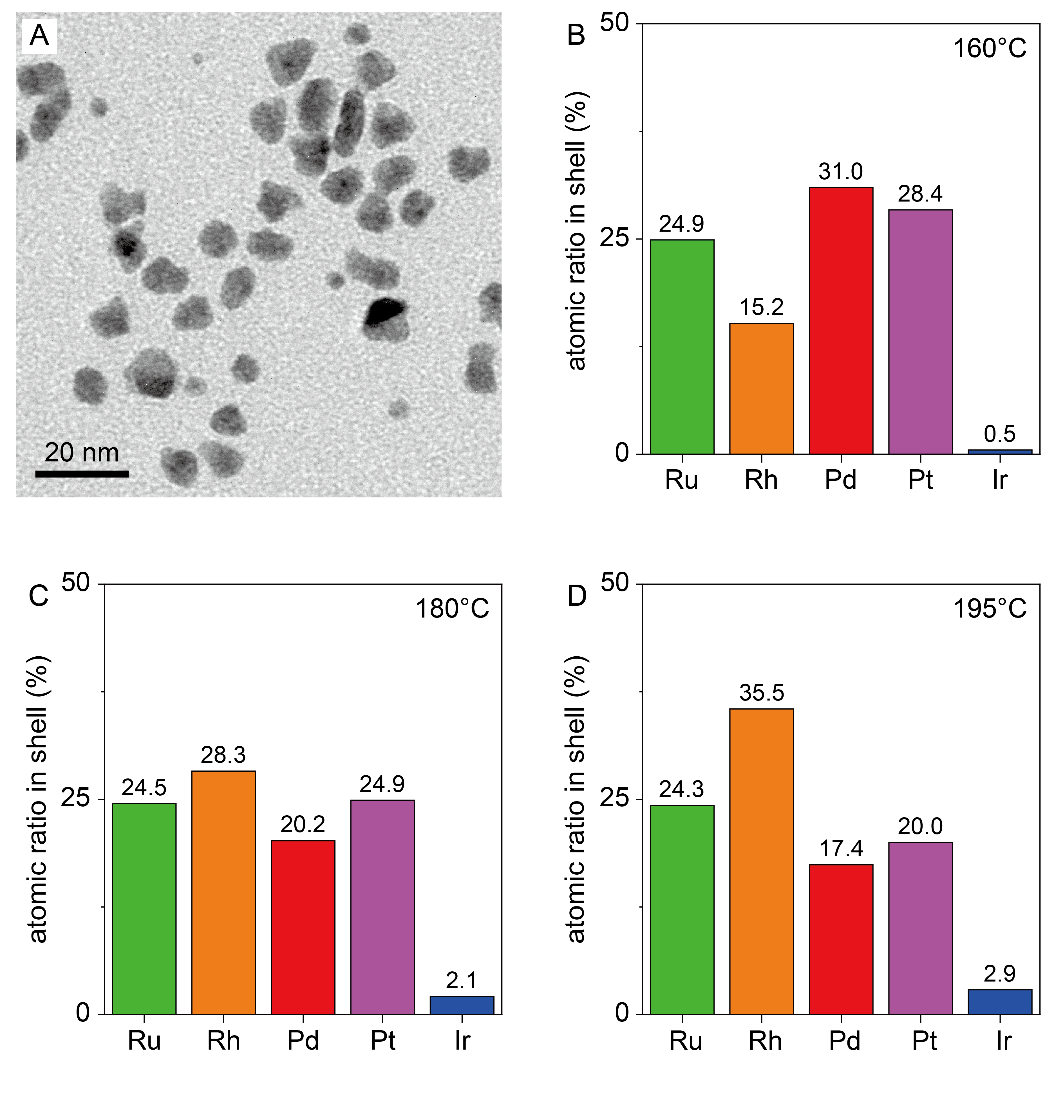


**Figure S2.** TEM image and ICP-OES analysis of Ru@RuRhPdPtIr core-shell nanocrystals synthesized using an equimolar mixture of Ru(acac)_3_, Rh(acac)_3_, Pd(acac)_2_, Pt(acac)_2_, and Ir(acac)_3_ as the precursor solution. (A) TEM image of Ru@RuRhPdPtIr synthesized at 160 °C. (B-D) ICP-OES analysis of RuRhPdPtIr shells synthesized at (B) 160 °C, (C) 180 °C, and (D) 195 °C. When synthesized at 160 °C, the Ir element is scarcely reduced. After elevating the reaction temperature to 180 °C and 195 °C, the atomic ratio of Ir remains approximately one-tenth of that of the other four elements, indicating the slow reduction kinetics of Ir(acac)_3_ compared to H_2_IrCl_6_·xH_2_O used (Figure 1J).

**
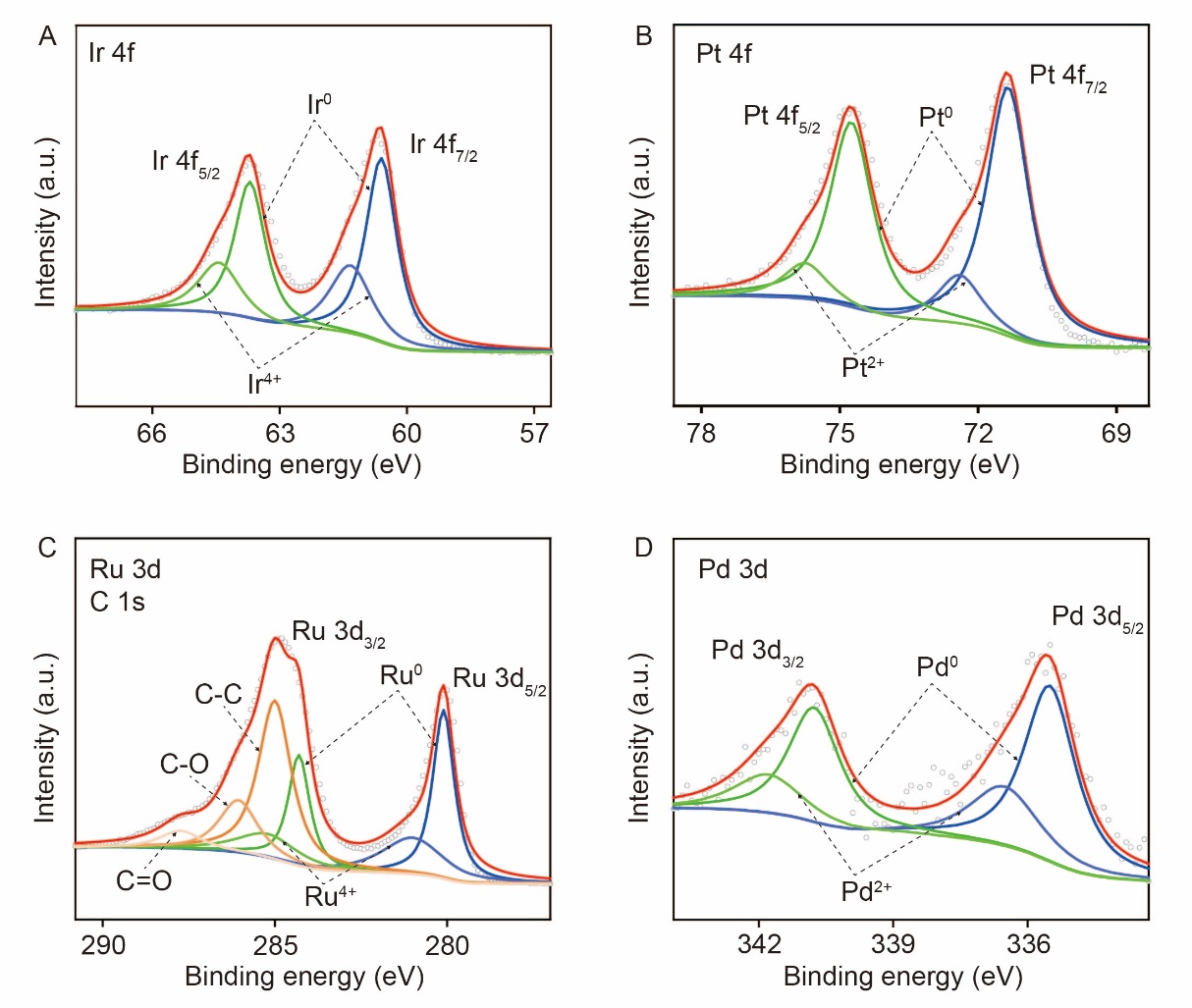
**

**Figure S3.** XPS spectra of Ru@Ru_0.2_Rh_0.2_Pd_0.2_Pt_0.2_Ir_0.2-4L_ core-shell nanocrystals. The spectra correspond to (A) Ir 4f, (B) Pt 4f, (C) Ru 3d, and (D) Pd 3d, depicting the valance states of each element. The XPS fitting results, presented in Figure S3A-D, reveal the presence of both major metallic and minor oxidized species in the Ir 4f, Pt 4f, Ru 3d, and Pd 3d peaks of the XPS spectra, which align well with the XANES fitting results outlined in Table 1. Additionally, in the 280-290 eV range, the C 1s signals indicate various chemical states, likely due to adsorbed PVP or organic contaminants from the environment. Although the Rh 3d signals overlap with Ir 4d and Pd 4d peaks in the 305-320 eV range, making it challenging to distinguish between metallic and oxidized Rh states through peak fitting, the XANES results further support that Rh primarily exists in its metallic form (Table 1). This conclusion is based on the significantly higher coordination number of Rh-4d and Rh-5d metallic bonds compared to Rh-O bonds, confirming that metallic Rh species dominate, with only minor oxidized species present.


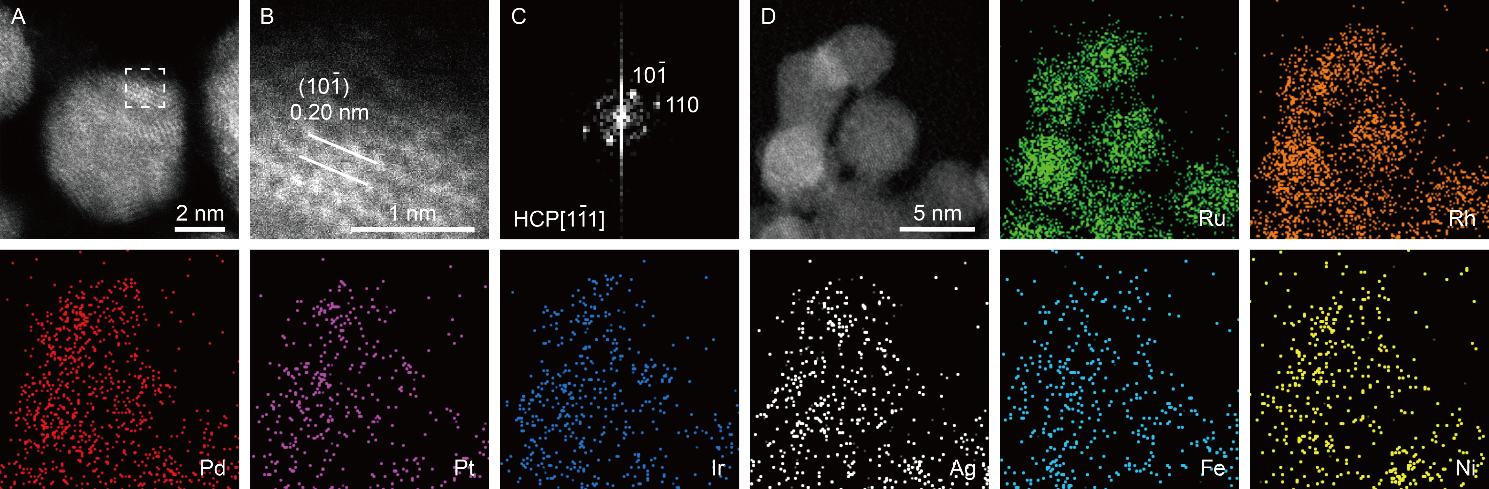


**Figure S4.** Structural and compositional characterization of Ru@RuRhPdPtIrAgFeNi core-shell nanocrystals. (A) HAADF-STEM image. (B) HAADF-STEM image enlarged from the white-dashed square in A. (C) FFT pattern from B. (D) EDS mappings. The HCP structure of Ru@RuRhPdPtIrAgFeNi is confirmed by the FFT diffraction pattern along the [1$\bar{\text{1}}$1] zone axis, and EDS mappings show the homogeneous distribution of Ru, Rh, Pd, Pt, Ir, Ag, Fe, and Ni on Ru cores.


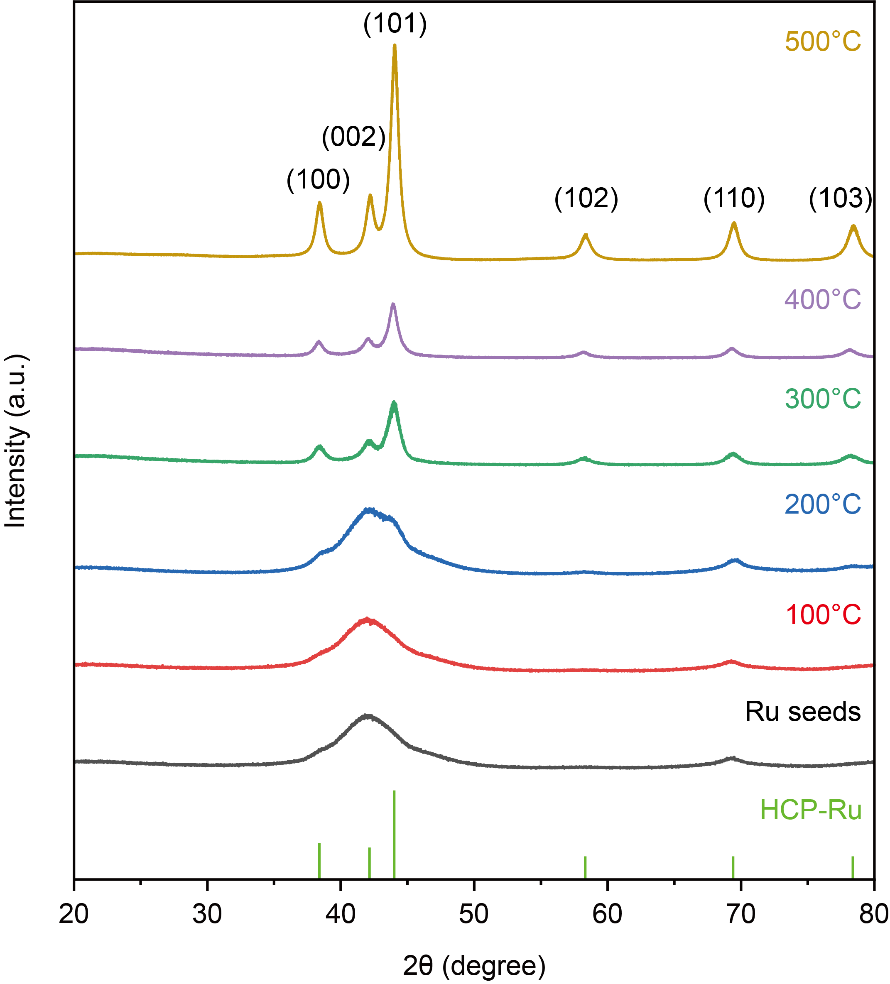


**Figure S5.** Synchrotron HRPXRD of Ru seeds with *in situ* heating from room temperature to 500 °C. The crystallization of Ru seeds increases with the elevation of temperature. The XRD standard card of HCP-Ru is PDF#06-0663.


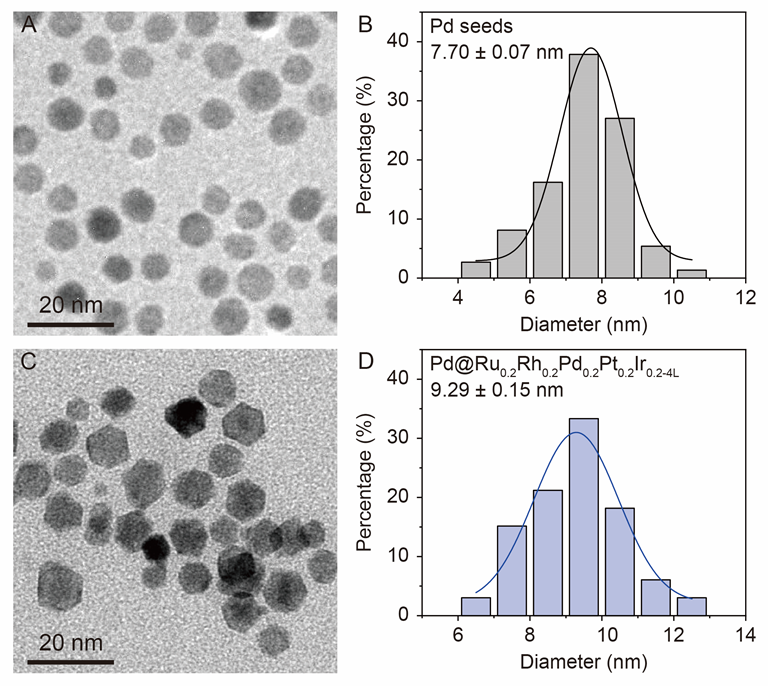


**Figure S6.** TEM images and size distributions of (A, B) Pd seeds, and (C, D) Pd@Ru_0.2_Rh_0.2_Pd_0.2_Pt_0.2_Ir_0.2-4L_ core-shell nanocrystals. The size of nanocrystals is measured using the ImageJ software.

**
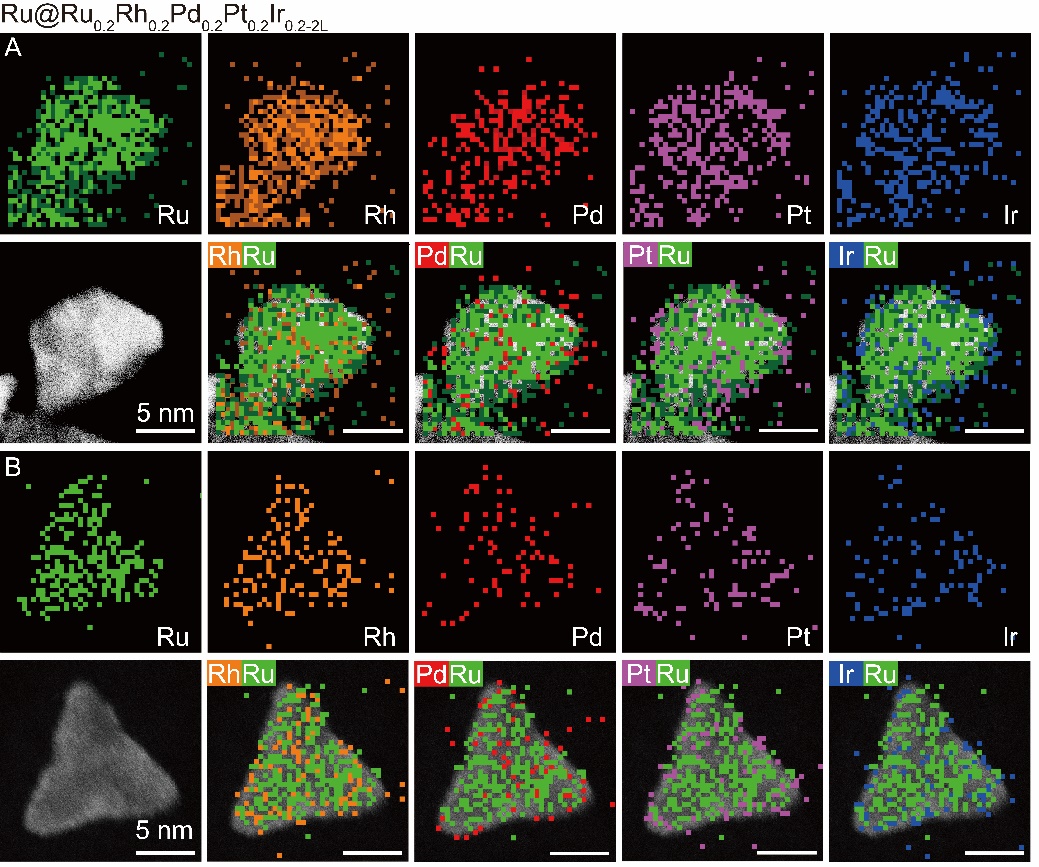
**

**Figure S7.** EDS mappings of HCP Ru@Ru_0.2_Rh_0.2_Pd_0.2_Pt_0.2_Ir_0.2-2L_ core-shell nanocrystals. (A) EDS mapping for each element of individual spherical-like nanocrystals, along with the corresponding mixed EDS mapping image showing the distribution of the seed (Ru) alongside each element (Rh, Pd, Pt, and Ir). (B) EDS mapping for each element of individual plate-like nanocrystals, along with the corresponding mixed EDS mapping image showing the distribution of the seed (Ru) alongside each element (Rh, Pd, Pt, and Ir). For Ru@Ru_0.2_Rh_0.2_Pd_0.2_Pt_0.2_Ir_0.2-2L_, the presence of only two atomic layers of HEA shells results in relatively weak signals. However, despite the weaker signal, all five element signals are roughly consistent with the shape observed in the EDS mappings, as confirmed by the mixed EDS mapping images. In addition, the ICP-OES analysis also indicates that the ratios of the elements in the HEA atomic layers for Ru@Ru_0.2_Rh_0.2_Pd_0.2_Pt_0.2_Ir_0.2-2L_ are 20.0%, 21.1%, 19.3%, 19.5%, and 20.1% for Ru, Rh, Pd, Pt, and Ir, respectively. These results suggest the successful formation of conformal HEA shells with equimolar ratios of the five elements.

**
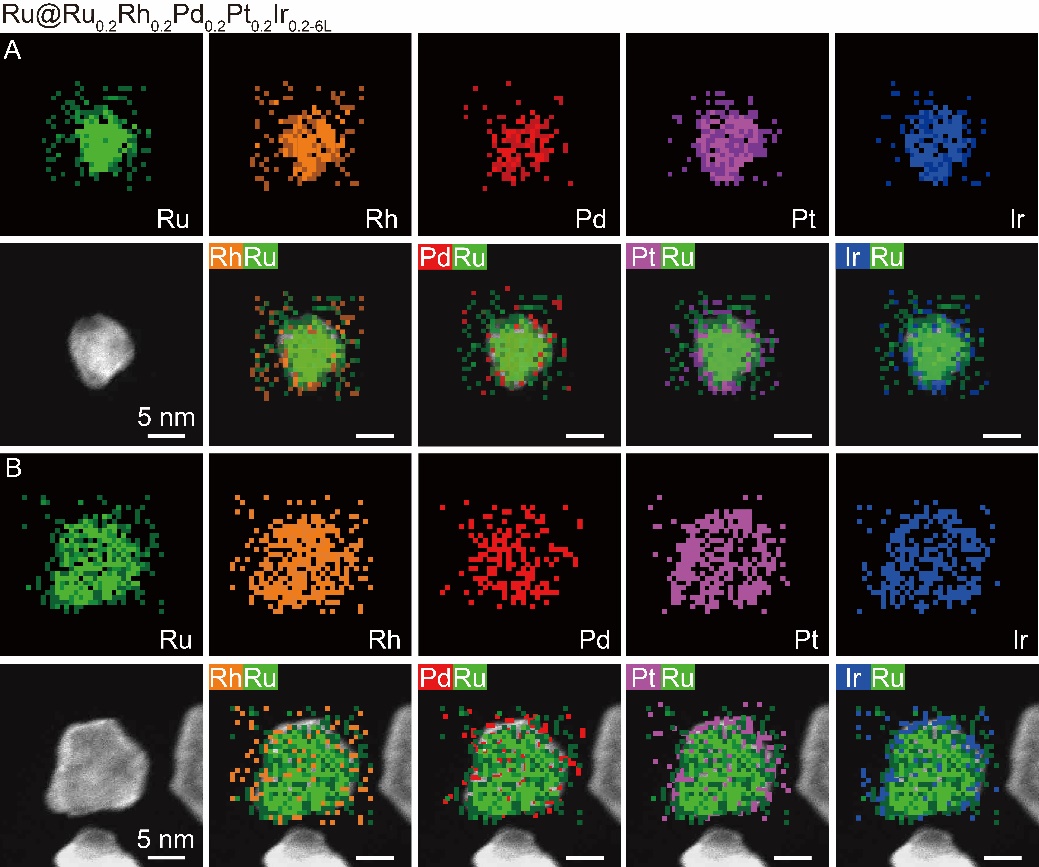
**

**Figure S8.** EDS mappings of HCP Ru@Ru_0.2_Rh_0.2_Pd_0.2_Pt_0.2_Ir_0.2-6L_ core-shell nanocrystals. (A) EDS mapping for each element of individual spherical-like nanocrystals, along with the corresponding mixed EDS mapping image showing the distribution of the seed (Ru) alongside each element (Rh, Pd, Pt, and Ir). (B) EDS mapping for each element of individual plate-like nanocrystals, along with the corresponding mixed EDS mapping image showing the distribution of the seed (Ru) alongside each element (Rh, Pd, Pt, and Ir).

**
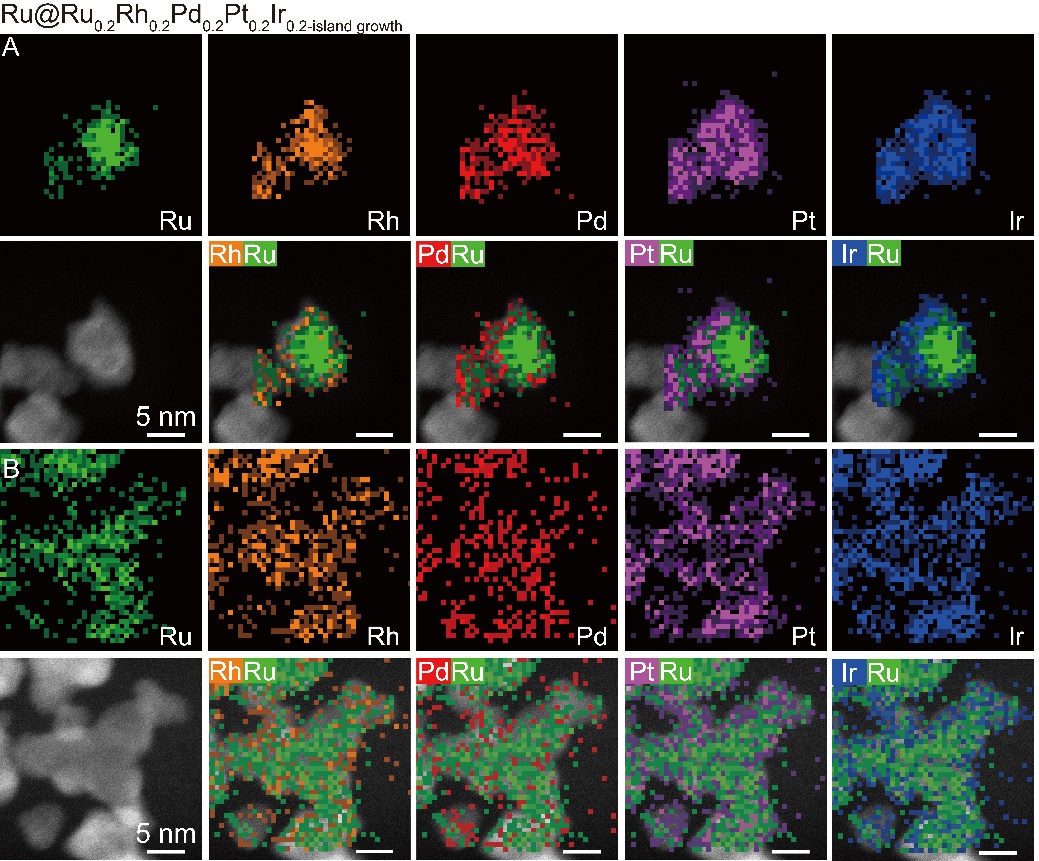
**

**Figure S9.** EDS mappings of HCP [Ru@Ru_0.2_Rh_0.2_Pd_0.2_Pt_0.2_Ir_0.2-island_](mailto:Ru@Ru0.2Rh0.2Pd0.2Pt0.2Ir0.2-island) _growth_ core-shell nanocrystals. (A) EDS mapping for each element of individual spherical-like nanocrystals, along with the corresponding mixed EDS mapping image showing the distribution of the seed (Ru) alongside each element (Rh, Pd, Pt, and Ir). (B) EDS mapping for each element of individual plate-like nanocrystals, along with the corresponding mixed EDS mapping image showing the distribution of the seed (Ru) alongside each element (Rh, Pd, Pt, and Ir).


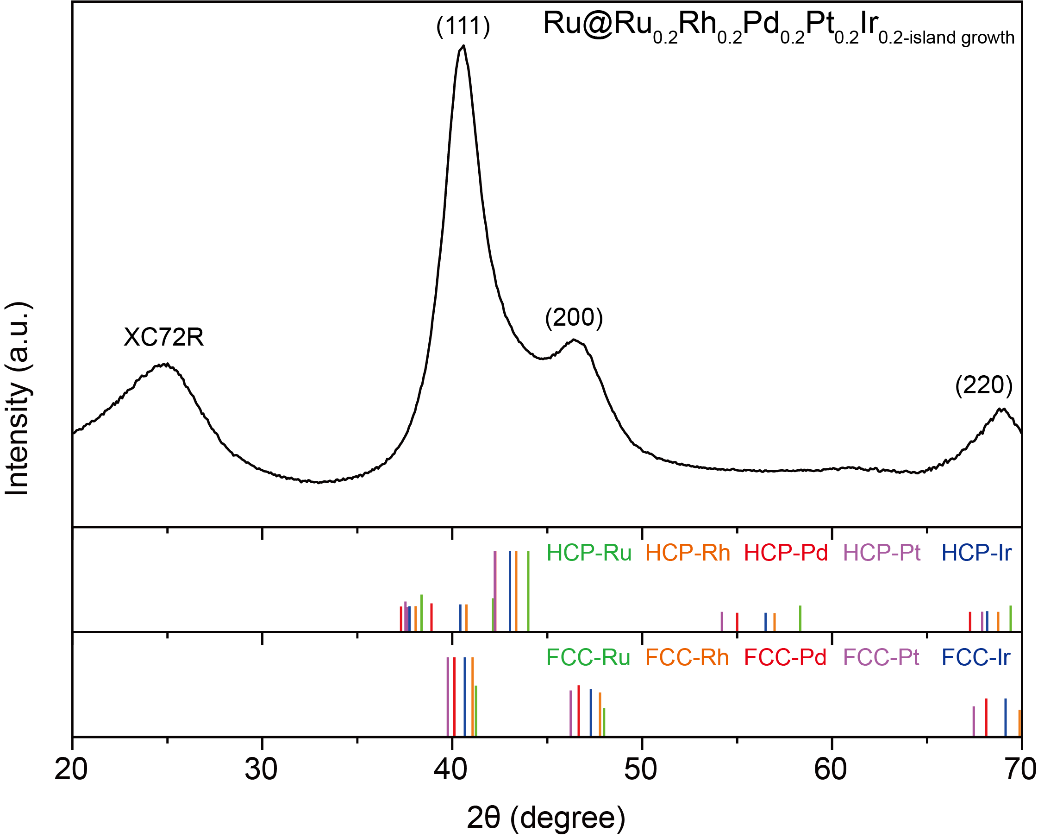


**Figure S10.** Synchrotron WAXS pattern of Ru@Ru_0.2_Rh_0.2_Pd_0.2_Pt_0.2_Ir_0.2-island growth_. The Ru@Ru_0.2_Rh_0.2_Pd_0.2_Pt_0.2_Ir_0.2-island growth_ nanocrystals are dispersed on the Vulcan XC72R for the WAXS measurement. The XRD standard cards of HCP-Ru, FCC-Rh, FCC-Pd, FCC-Pt, and FCC-Ir are PDF#06-0663, PDF#05-0685, PDF#46-1043, PDF#05-0685, and PDF#46-1044, respectively. The standard cards of HCP-Rh, HCP-Pd, HCP-Pt, HCP-Ir, and FCC-Ru are derived from the open database “Materials Project”.

**
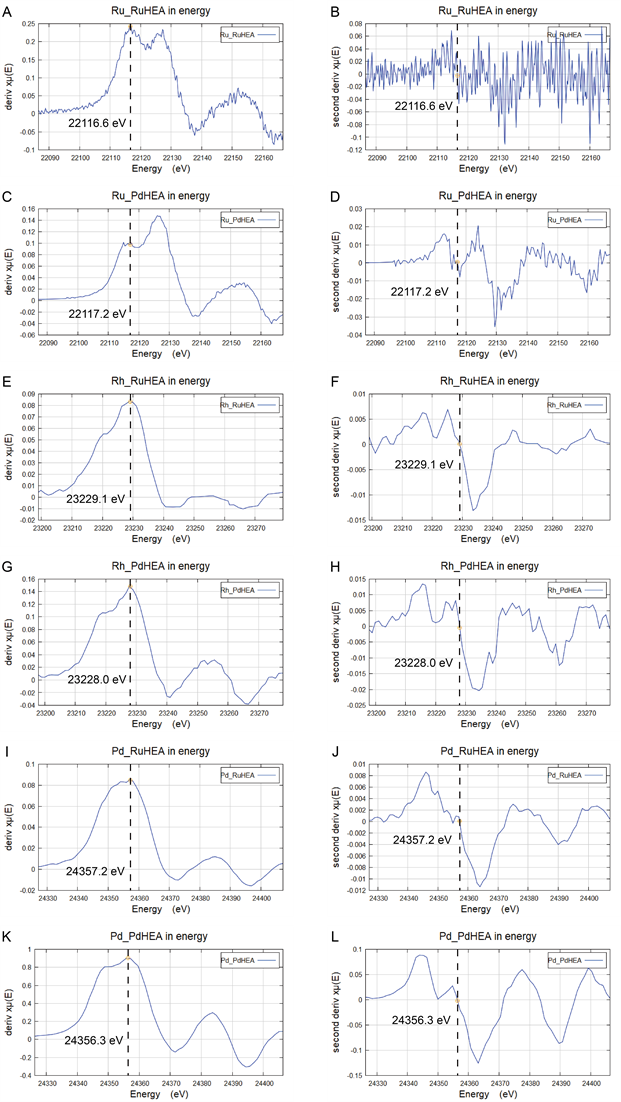
**

**Figure S11.** The Ru, Rh, and Pd K-edge absorption positions determined by the inflection points of pre-edges for HCP Ru@Ru_0.2_Rh_0.2_Pd_0.2_Pt_0.2_Ir_0.2-4L_ and FCC Pd@Ru_0.2_Rh_0.2_Pd_0.2_Pt_0.2_Ir_0.2-4L_. (A-D) Ru K-edge, (E-H) Rh K-edge, and (I-L) Pd K-edge. The data are acquired by using “Athena software” and the energy of absorption edge is calibrated by the reference metallic foil corresponding to each element. The inflection points of pre-edges are identified where the second derivative equals to 0.


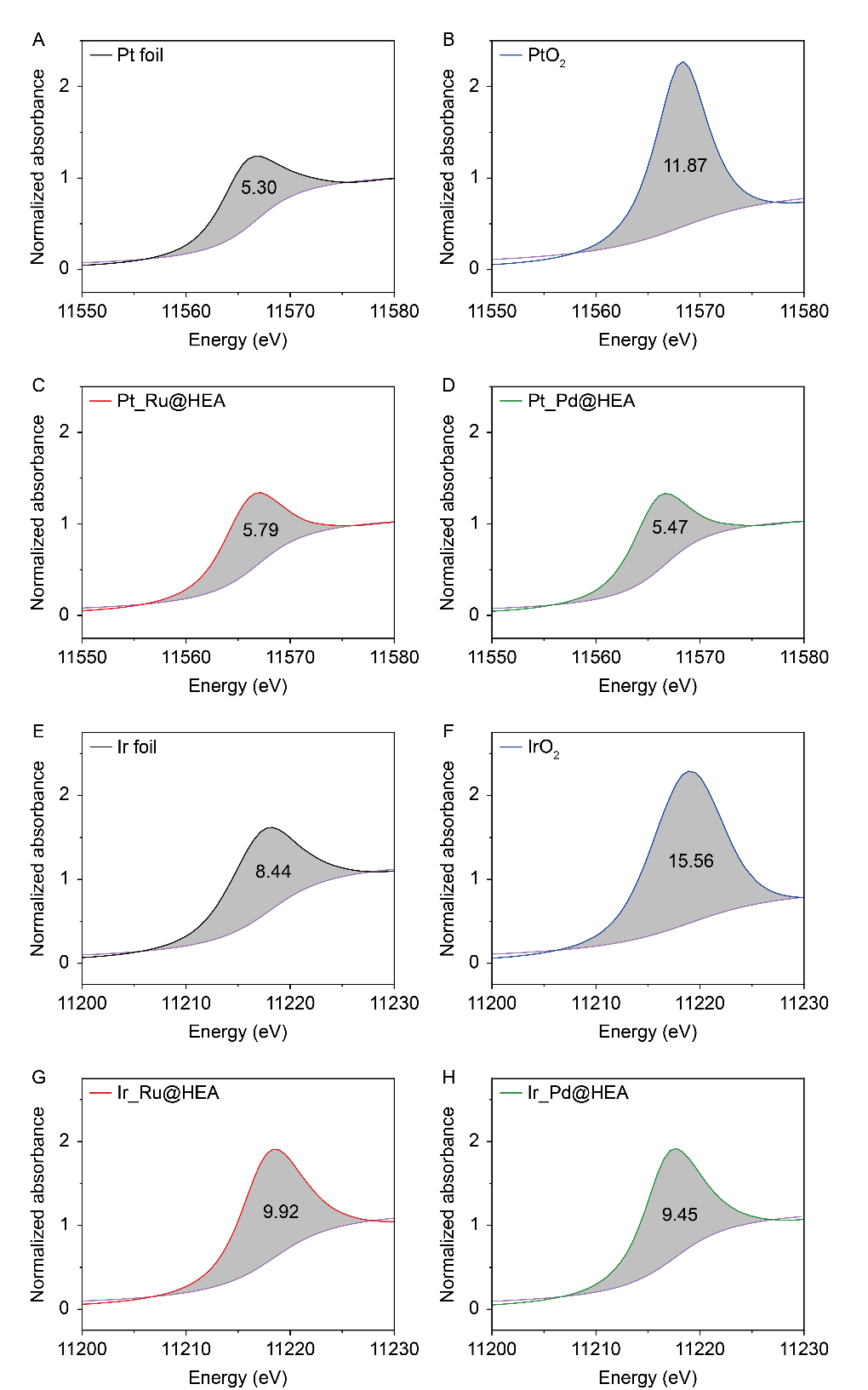


**Figure S12.** White-line peak areas of Pt L_3_-edge and Ir L_3_-edge for HCP Ru@Ru_0.2_Rh_0.2_Pd_0.2_Pt_0.2_Ir_0.2-4L_, FCC Pd@Ru_0.2_Rh_0.2_Pd_0.2_Pt_0.2_Ir_0.2-4L_, metallic foils, and oxides. The peak area is extracted by subtracting the arctangent function background.^[5]^ The center of the arctangent background is set at the white-line peak position, using the “peak fitting” function in “Athena software”.


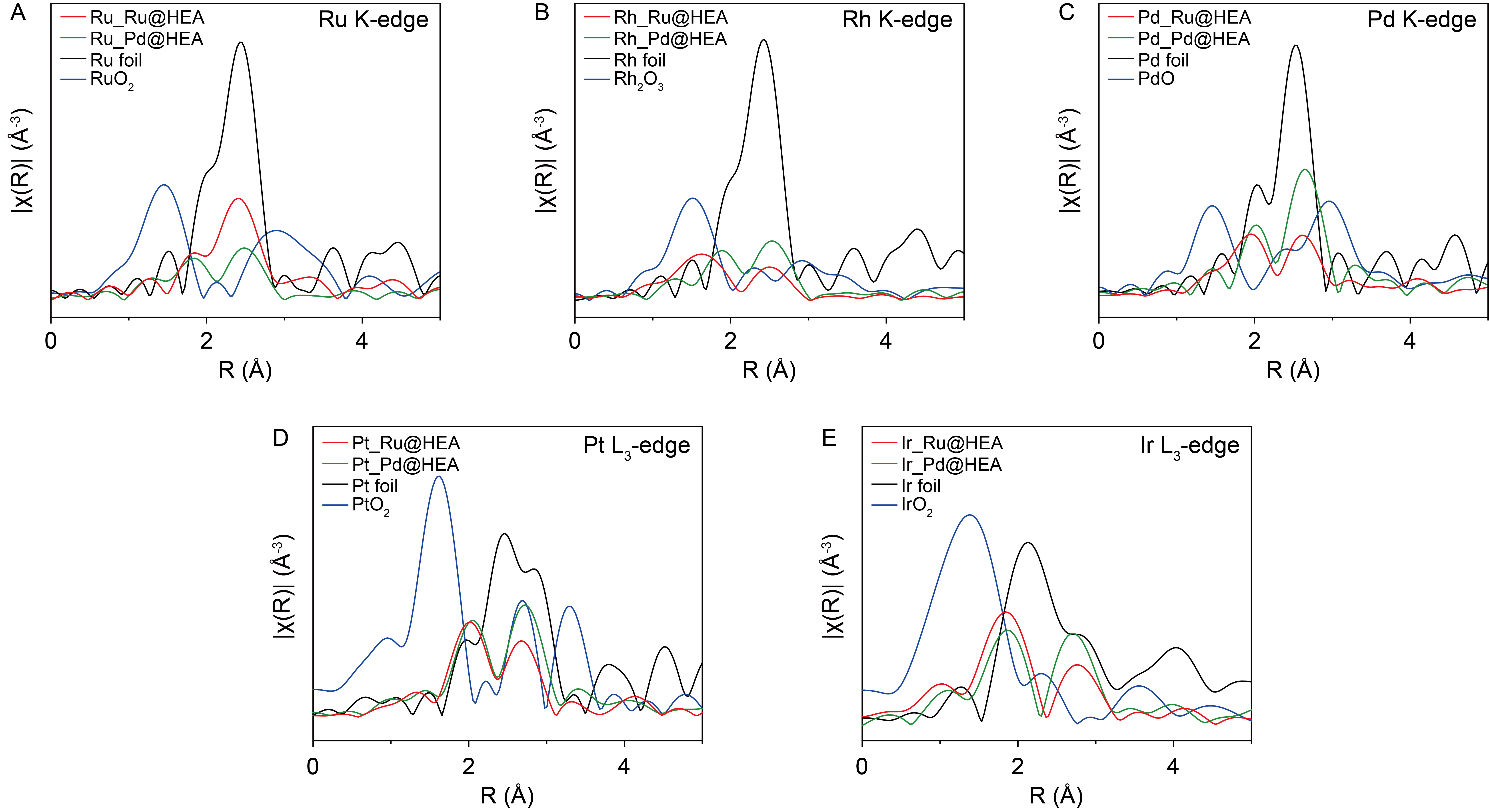


**Figure S13.** FT-EXAFS spectra of HCP Ru@Ru_0.2_Rh_0.2_Pd_0.2_Pt_0.2_Ir_0.2-4L_ and FCC Pd@Ru_0.2_Rh_0.2_Pd_0.2_Pt_0.2_Ir_0.2-4L_ along with the corresponding metallic foils and oxides. (A) Ru K-edge. (B) Rh K-edge. (C) Pd K-edge. (D) Pt L_3_-edge. (D) Ir L_3_-edge.


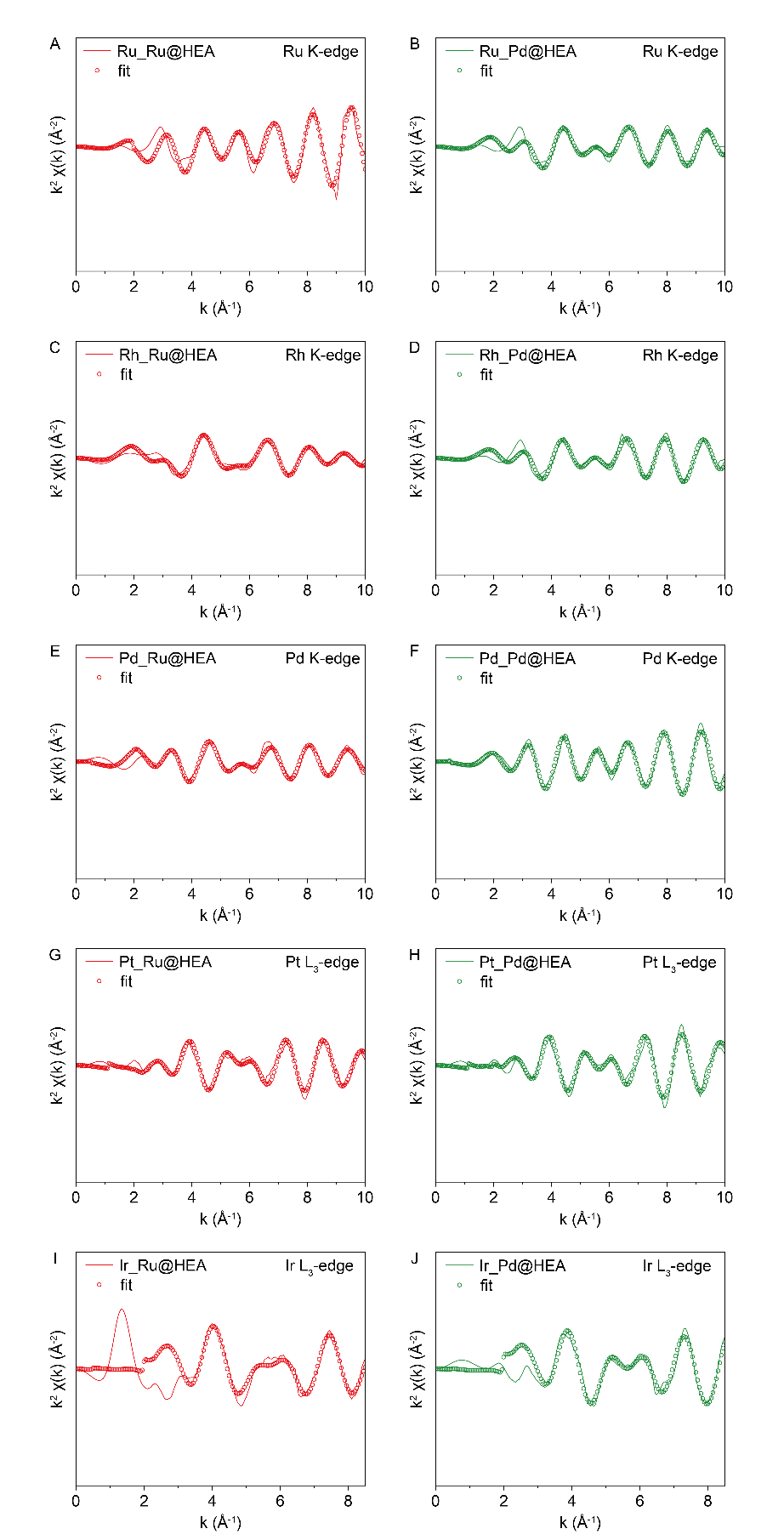


**Figure S14.** EXAFS spectra in k-space along with the fitting results for HCP Ru@Ru_0.2_Rh_0.2_Pd_0.2_Pt_0.2_Ir_0.2-4L_ and FCC Pd@Ru_0.2_Rh_0.2_Pd_0.2_Pt_0.2_Ir_0.2-4L_. (A, B) Ru K-edge. (C, D) Rh K-edge. (E, F) Pd K-edge. (G, H) Pt L_3_-edge. (I, J) Ir L_3_-edge.


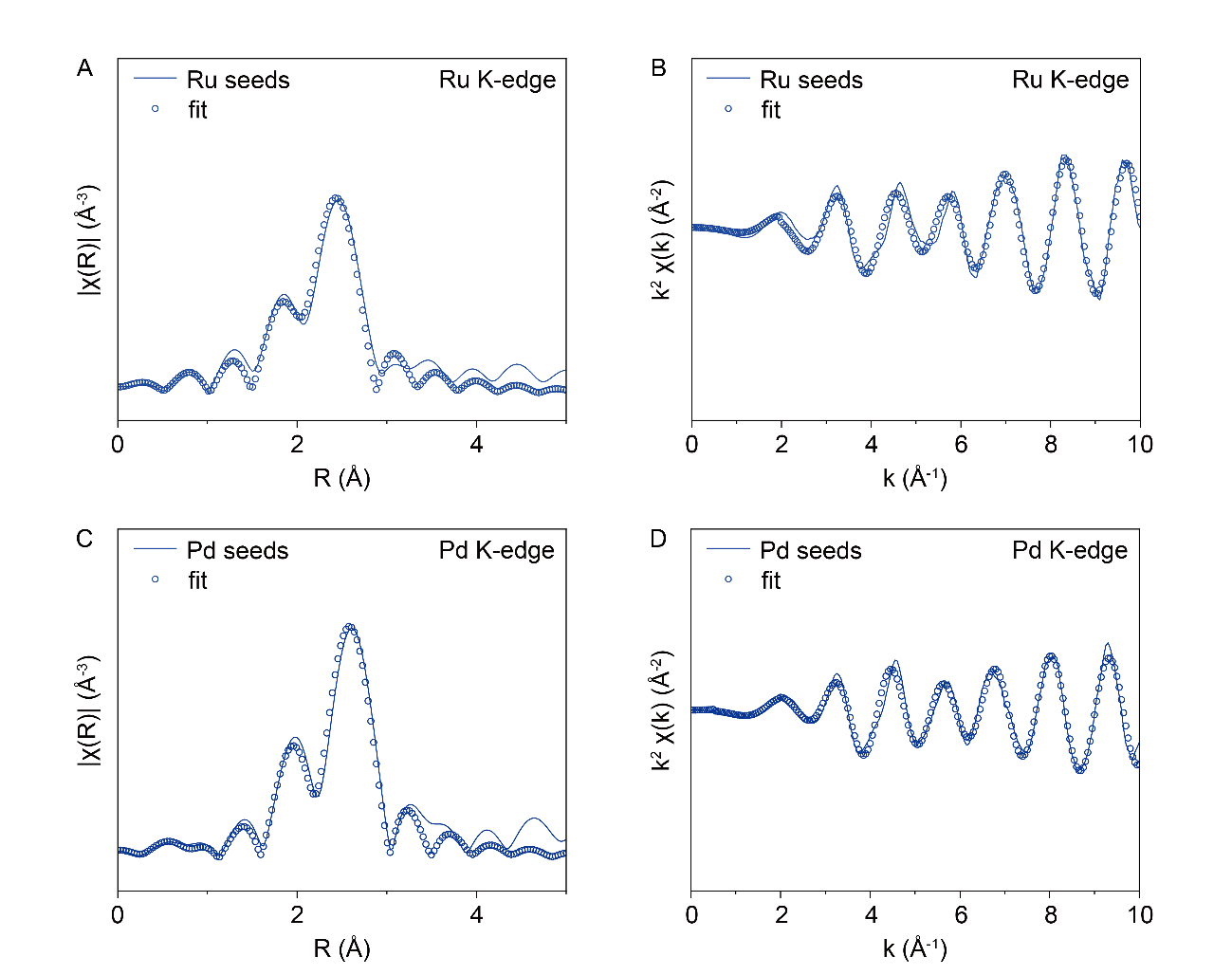


**Figure S15.** EXAFS spectra in R-space and k-space along with the fitting results for Ru seeds and Pd seeds. (A, B) Ru seeds in Ru K-edge. (C, D) Pd seeds in Pd K-edge.


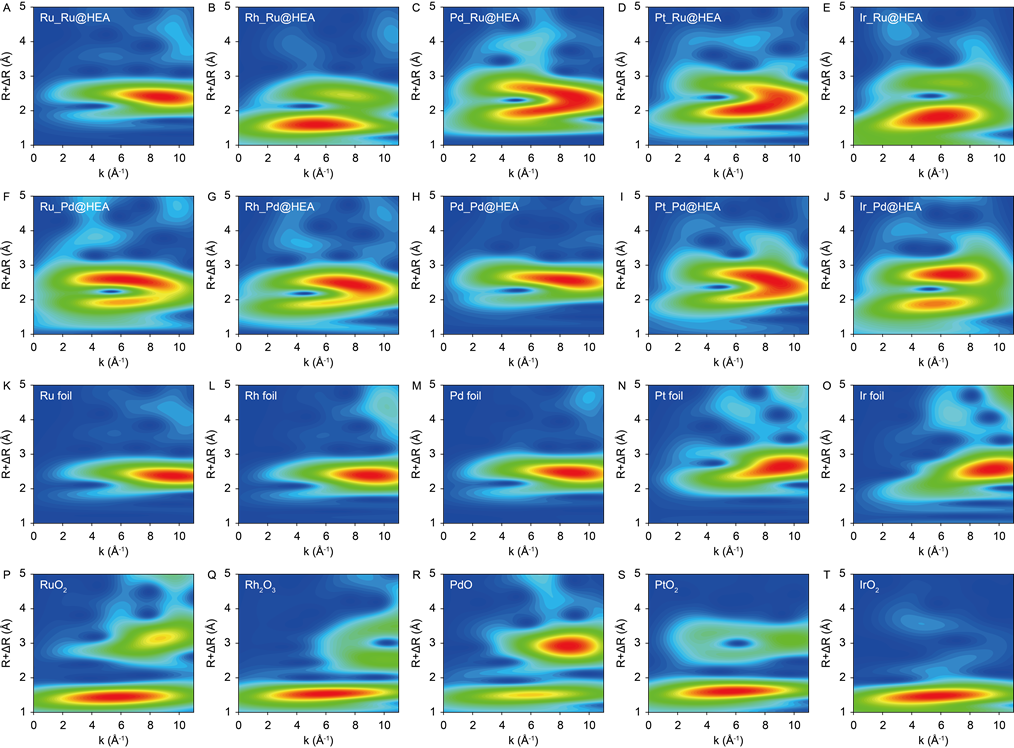


**Figure S16.** WT-EXAFS contour maps of HCP Ru@Ru_0.2_Rh_0.2_Pd_0.2_Pt_0.2_Ir_0.2-4L_, FCC Pd@Ru_0.2_Rh_0.2_Pd_0.2_Pt_0.2_Ir_0.2-4L_, metallic foils and oxides. (A-E) Ru@Ru_0.2_Rh_0.2_Pd_0.2_Pt_0.2_Ir_0.2-4L_ from (A) Ru K-edge, (B) Rh K-edge, (C) Pd K-edge, (D) Pt L_3_-edge, and (E) Ir L_3_-edge. (F-J) Pd@Ru_0.2_Rh_0.2_Pd_0.2_Pt_0.2_Ir_0.2-4L_ from (F) Ru K-edge, (G) Rh K-edge, (H) Pd K-edge, (I) Pt L_3_-edge, and (J) Ir L_3_-edge. (K) Ru foil. (L) Rh foil. (M) Pd foil. (N) Pt foil. (O) Ir foil. (P) RuO_2_. (Q) Rh_2_O_3_. (R) PdO. (S) PtO_2_. (T) IrO_2_.


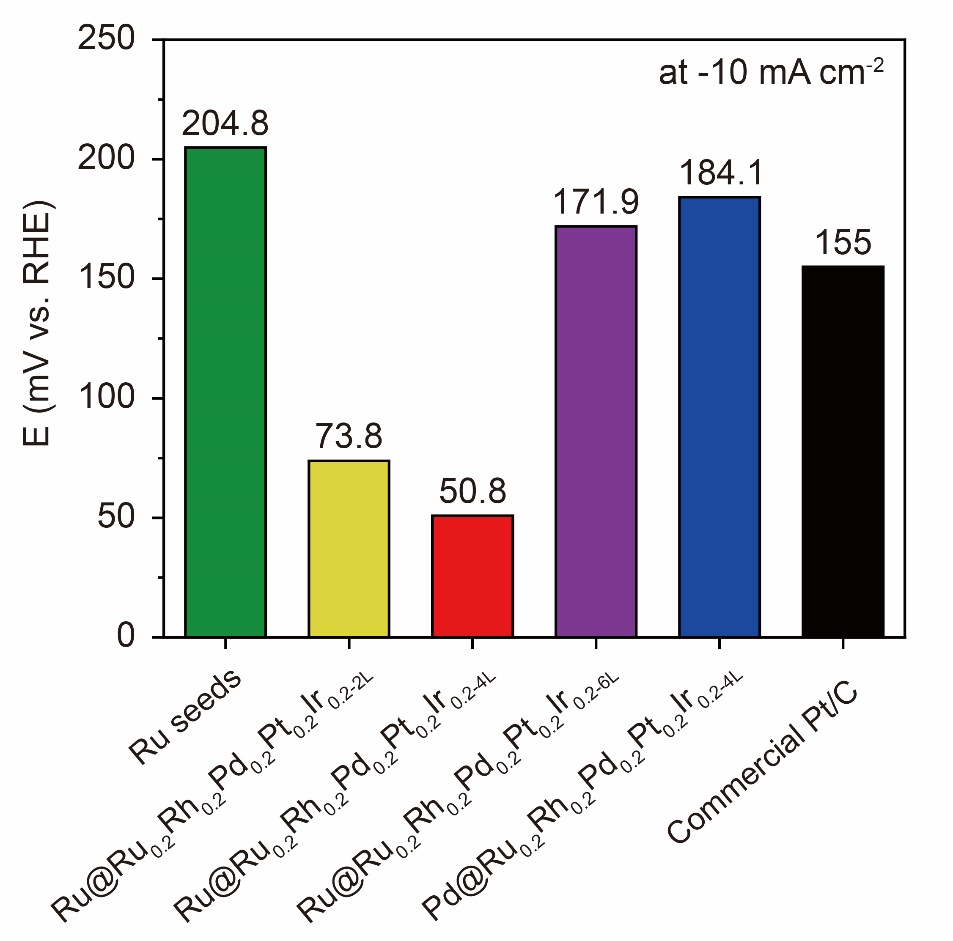


**Figure S17.** Overpotentials of Ru seeds, HCP Ru@Ru_0.2_Rh_0.2_Pd_0.2_Pt_0.2_Ir_0.2-nL_, FCC Pd@Ru_0.2_Rh_0.2_Pd_0.2_Pt_0.2_Ir_0.2-4L_ core-shell nanocrystals, and commercial Pt/C in alkaline HER at -10 mA cm^-2^.


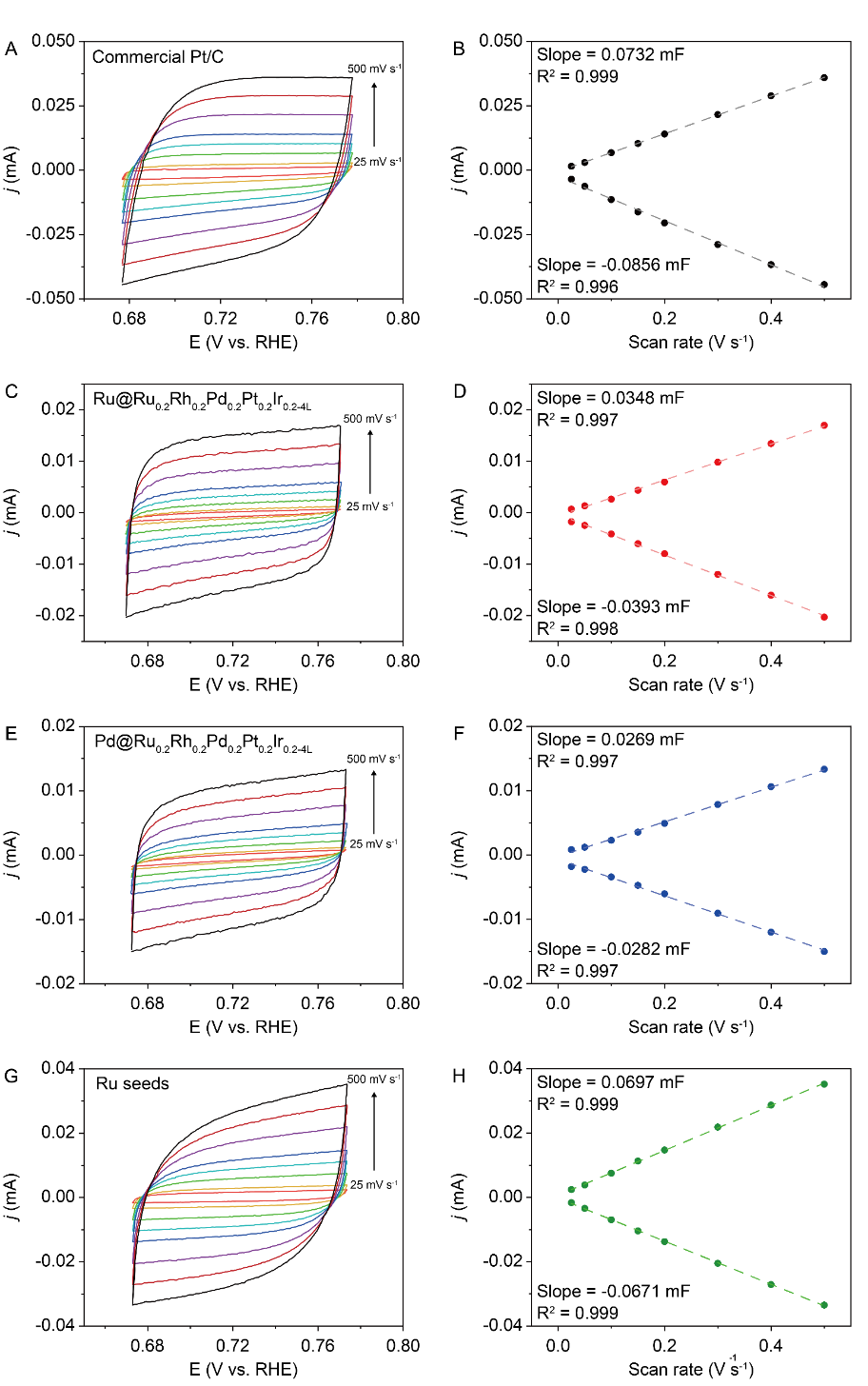


**Figure S18.** Double layer capacitance (C_dl_) of commercial Pt/C, Ru@Ru_0.2_Rh_0.2_Pd_0.2_Pt_0.2_Ir_0.2-4L_, Pd@Ru_0.2_Rh_0.2_Pd_0.2_Pt_0.2_Ir_0.2-4L_, and Ru seeds determined by cyclic voltammograms in 0.5 M H_2_SO_4_. CV curves are derived at the scan rate from 25 mV s^-1^ to 500 mV s^-1^. The ECSA is calculated by ECSA = C_dl_/C_s_ (C_s_ = specific capacitance = 40 μF cm^-2^).^[6]^ The C_dl_ of commercial Pt/C, Ru@Ru_0.2_Rh_0.2_Pd_0.2_Pt_0.2_Ir_0.2-4L_, Pd@Ru_0.2_Rh_0.2_Pd_0.2_Pt_0.2_Ir_0.2-4L_, and Ru seeds are 79.4 μF, 37.05 μF, 27.55 μF and 68.4 μF, respectively. Thus, the ECSA are 1.985 cm^2^, 0.926 cm^2^, 0.689 cm^2^, and 1.71 cm^2^ for commercial Pt/C, Ru@Ru_0.2_Rh_0.2_Pd_0.2_Pt_0.2_Ir_0.2-4L_, Pd@ Ru_0.2_Rh_0.2_Pd_0.2_Pt_0.2_Ir_0.2-4L_, and Ru seeds, respectively.


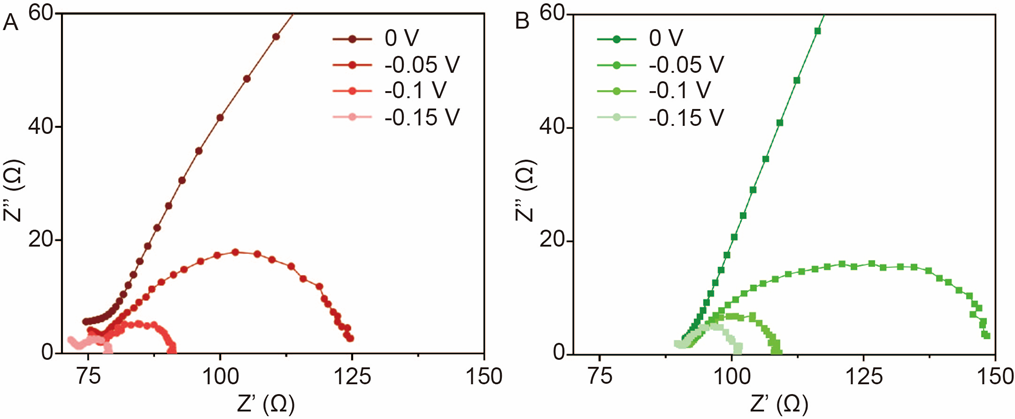


**Figure S19.** Nyquist plots for (A) [Ru@Ru_0.2_Rh_0.2_Pd_0.2_Pt_0.2_Ir_0.2-4L_](mailto:Ru@Ru0.2Rh0.2Pd0.2Pt0.2Ir0.2-4L), and (B) commercial Pt/C under different overpotentials of 0, -0.05, -0.1, and -0.15 V vs RHE.


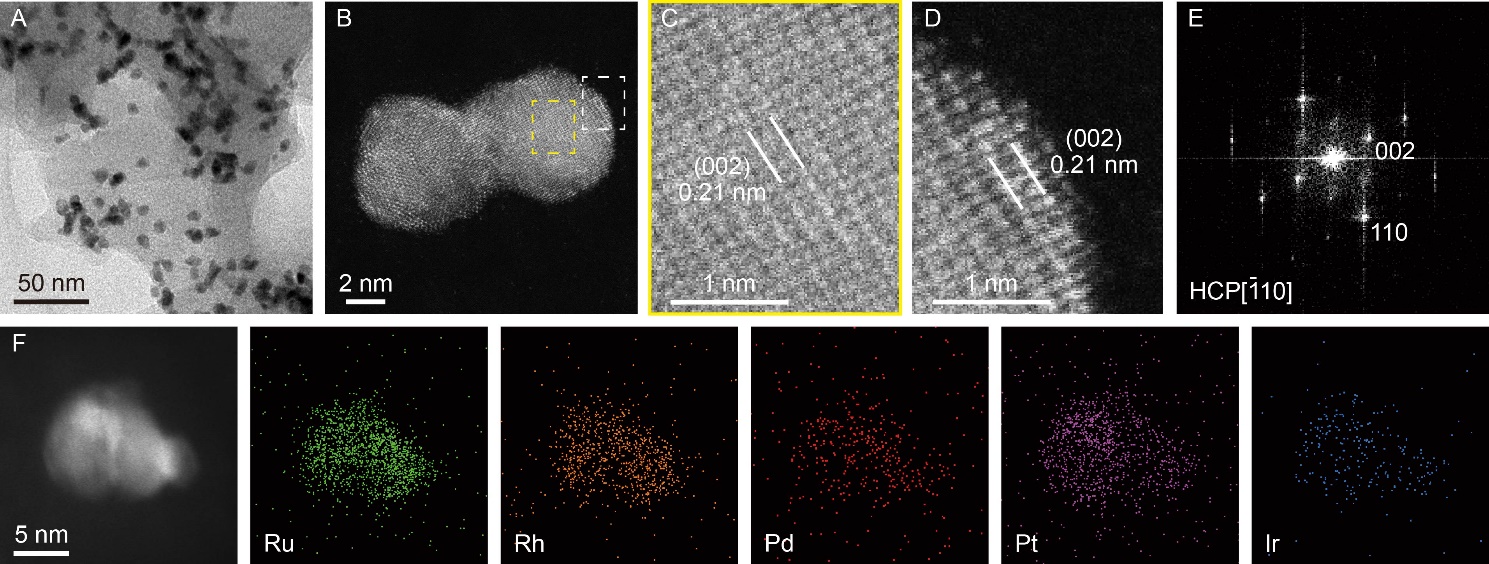


**Figure S20.** Structural and compositional characterization of Ru@Ru_0.2_Rh_0.2_Pd_0.2_Pt_0.2_Ir_0.2-4L_ dispersed on the XC72R carbon black after 15,000 cycles alkaline HER durability test. (A) TEM, (B) HAADF-STEM, (C, D) Enlarged HAADF-STEM images (E) FFT images of (B), and (F) EDS mapping of Ru@Ru_0.2_Rh_0.2_Pd_0.2_Pt_0.2_Ir_0.2-4L_ after the durability test. The FFT pattern in (E) indicates the HCP crystal structure. The atomic-resolution HAADF-STEM image and the corresponding FFT pattern reveal that the individual nanocrystal retains the characteristic atomic arrangement when observed along the HCP [$\bar{\text{1}}$10] zone axis. The lattice distance for both the Ru cores and the RuRhPdPtIr (002) plane is measured to be around approximately 0.21 nm. These findings confirm that the HEA shell layers retain their HCP structure after long-term electrocatalytic reactions.


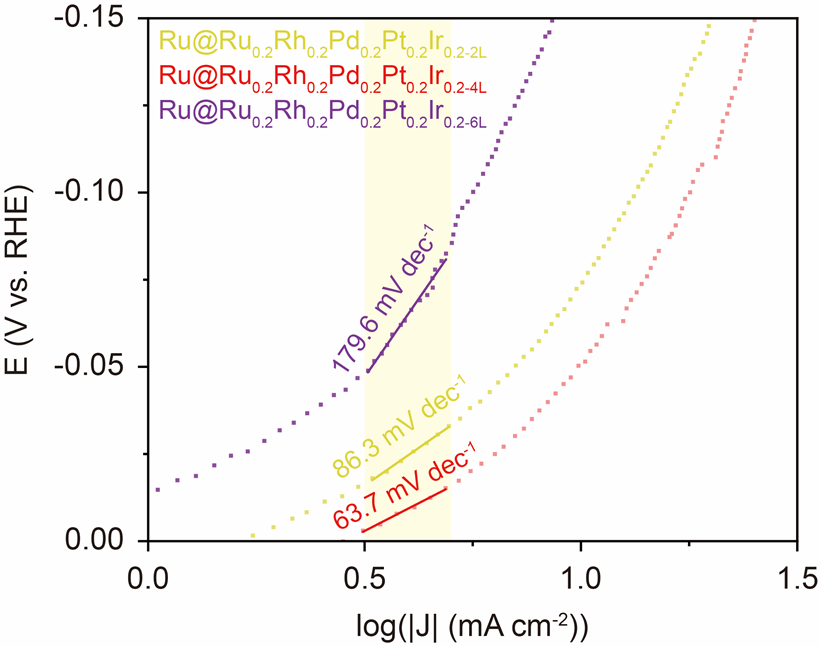


**Figure S21.** Tafel plots of HCP [Ru@Ru_0.2_Rh_0.2_Pd_0.2_Pt_0.2_Ir_0.2-2L_](mailto:Ru@Ru0.2Rh0.2Pd0.2Pt0.2Ir0.2-2L), [Ru@Ru_0.2_Rh_0.2_Pd_0.2_Pt_0.2_Ir_0.2-4L_](mailto:Ru@Ru0.2Rh0.2Pd0.2Pt0.2Ir0.2-2L), and [Ru@Ru_0.2_Rh_0.2_Pd_0.2_Pt_0.2_Ir_0.2-6L_](mailto:Ru@Ru0.2Rh0.2Pd0.2Pt0.2Ir0.2-6L) core-shell nanocrystals. The corresponding Tafel slopes for these samples are 86.3 mV dec^-1^, 63.7 mV dec^-1^, and 179.6 mV dec^-1^, respectively.


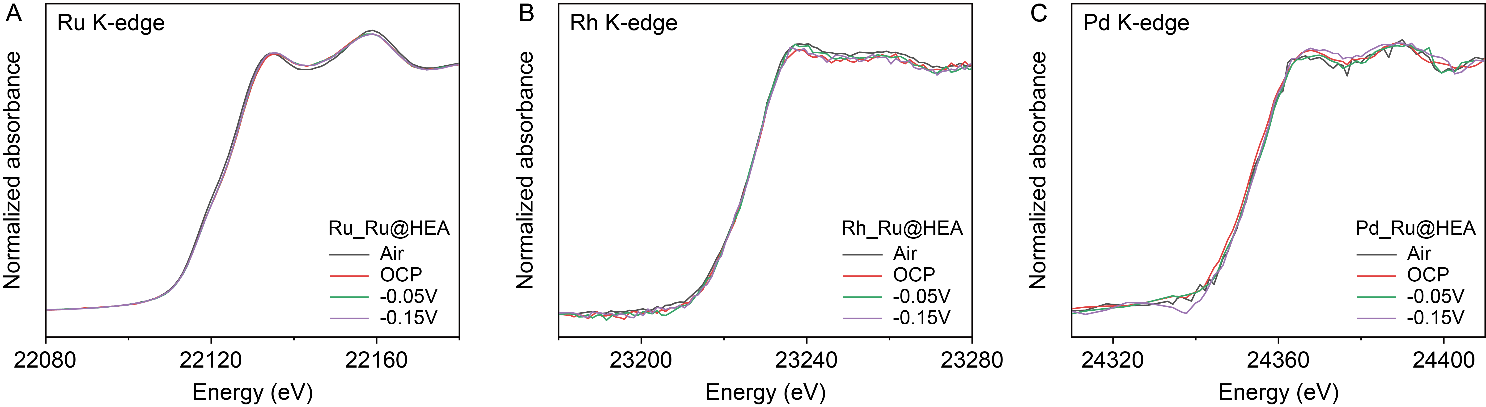


**Figure S22.** *Operando* synchrotron XAS measurements of Ru@Ru_0.2_Rh_0.2_Pd_0.2_Pt_0.2_Ir_0.2-4L_ under alkaline HER. XANES spectra of (A) Ru K-edge, (B) Rh K-edge, and (C) Pd K-edge.


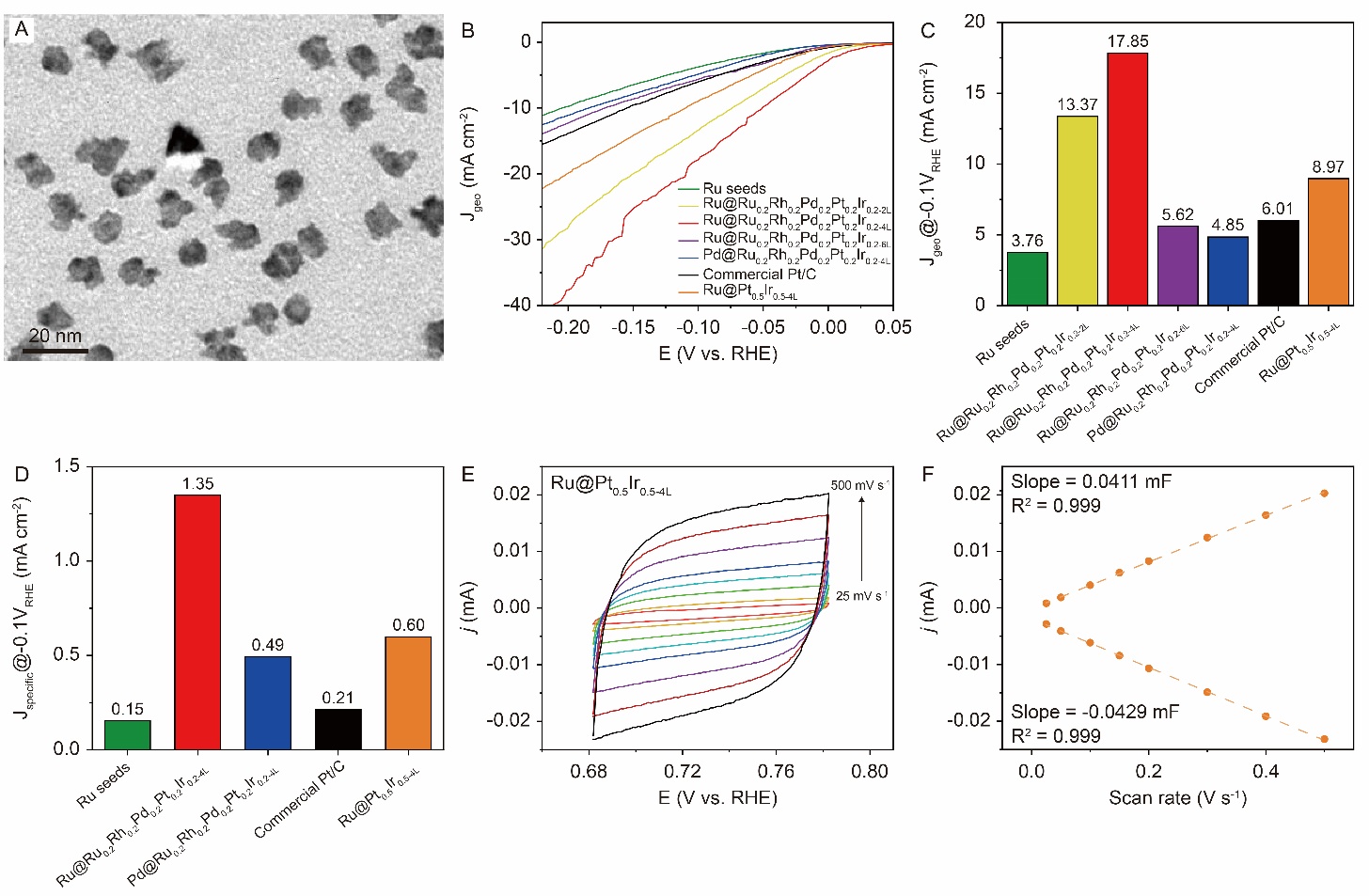


**Figure S23.** Structural characterization and electrochemical measurements of Ru@Pt_0.5_Ir_0.5-4L_ core-shell nanocrystals. (A) TEM image. (B) Polarization curves of alkaline HER in 1.0 M KOH. (C) Current density at -0.1 V_RHE_. (D) Specific activities at -0.1 V_RHE_. (E, F) Cyclic voltammograms for ECSA determination (C_dl_ = 42 μF, ECSA = C_dl_/C_s_ = 1.05 cm^2^). The Ru@Pt_0.5_Ir_0.5-4L_ show similar surface area as compared to [Ru@Ru_0.2_Rh_0.2_Pd_0.2_Pt_0.2_Ir_0.2-4_](mailto:Ru@Ru0.2Rh0.2Pd0.2Pt0.2Ir0.2-4)_L_ (0.926 cm^2^).

**Computational Details**

To simulate the properties of a RuRhPdPtIr HEA layers, we construct a 3x3, four-layer hcp (100) slab due to its lower surface energy and greater abundance in metal particles.^[7, 8]^ The process begins by optimizing the structures of each constituent metal (Ru, Rh, Pd, Pt, and Ir). From these optimized structures, we calculate the individual lattice constants for each metal. These constants are then converted into their respective metallic radii. By averaging the radii, we obtain a mean radius (r_ave_), which serves as the basis for determining the final lattice constant.^[9]^ This methodology ensures an accurate representation of the mixed-metal characteristics in the model. To explore adsorption phenomena, we generate 10 unique RuRhPdPtIr slabs to study both hydrogen and hydroxide adsorption.

H-adsorption sites

Each RuRhPdPtIr hcp (100) slab contains 15 hollow hydrogen adsorption sites, resulting in a total of 150 potential adsorption positions. Starting from these 150 initial H-adsorption configurations, we performed geometric optimizations for each, observing that several converged to identical adsorption states, thereby reducing the number of unique configurations to 80. This optimization ensures that we focus on energetically favorable configurations without redundancy.

The adsorption free energy of hydrogen (∆*G*_H*_) serves as a crucial indicator of catalytic efficiency in the HER. Previous studies have shown that when ∆*G*_H*_ approaches zero, the reaction conditions become more favorable, promoting efficient HER catalysis*.*^[10, 11]^

To assess ∆*G*_H*_, we apply the following equation:

∆*G*_H*_ **=** *G*(H*) **−** *G*(*) **−** ½ *G*(H_2_) **≈** E (H*) **−** *E*(*) **−** ½ *E*(H_2_) **+** 0.24 (in *e*V)

In this equation, E (H*) represents the electronic energy of the slab with hydrogen adsorbed, and *E* (*) is the electronic energy of the slab without hydrogen. The value 0.24 eV accounts for zero-point energy corrections and entropy effects, which refine the accuracy of the energy estimation. This calculation helps us evaluate the catalytic efficiency of different materials in the HER. By determining how close ∆*G*_H*_ is to zero, we can identify materials with favorable reaction conditions, ultimately guiding the development of more effective catalysts.


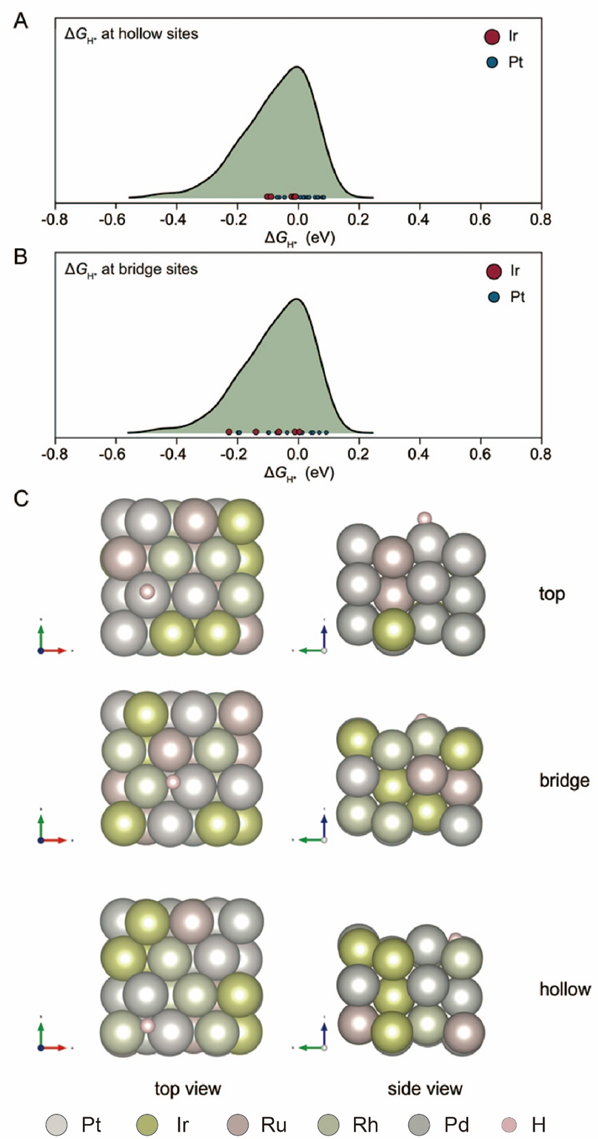


**Figure S24.** H-adsorption behaviors on selected sites, and the H-adsorption configurations of RuRhPdPtIr HEA layers. (A) ∆*G*_H*_ at hollow sites where Ir and Pt are present. (B) ∆*G*_H*_ at bridge sites where Ir and Pt are present. (C) H-adsorption configurations of RuRhPdPtIr hcp (100) surface. In the RuRhPdPtIr hcp (100) surface, Pt shows optimal H-adsorption free energy as compared to Ir in both hollow and bridge sites ((A) and (B)). This trend is also observed in the adsorption of hydrogen on the top sites (Figure 7F).

OH-adsorption sites

While hydrogen adsorption is a crucial factor in evaluating catalytic performance in the HER, the role of hydroxide (OH) adsorption is equally important and cannot be overlooked. By examining both hydrogen and hydroxide adsorption, we can obtain a more holistic view of the behavior of the catalyst during the reaction, providing deeper insights into its potential efficiency.

For the calculation of OH adsorption energy, we select 12 top sites on each RuRhPdPtIr hcp (100) surface, initially identifying 120 possible adsorption configurations. However, following geometric optimization, several configurations converge, resulting in 85 unique adsorption states. This optimization process, much like the one used for hydrogen adsorption, allows us to narrow our focus to the most energetically favorable configurations. Previous research has provided a framework for calculating OH adsorption free energy*.*^[12]^

To assess ∆*G*_OH*_, we apply the following equation:

∆*G*_OH*_ **=** *G*(OH*) **−** *G*(*) **–** [*G*(H_2_O) **−** ½ *G*(H_2_)] **≈** E (OH*) **−** *E*(*) **– [**E (H_2_O) **−** ½ *E*(H_2_)] **+** 0.29 (in *e*V)

In this equation, E (OH*) refers to the electronic energy of the surface with adsorbed hydroxide, and *E*(***) represents the energy of the clean surface. The term **[**E (H_2_O) ***−*** ½ *E*(H_2_)] accounts for the energies of water and hydrogen molecules, with 0.29 eV included to account for zero-point energy corrections, solvation energy, and entropy effects. This method, widely adopted in previous studies, provides an efficient way to estimate the free energy of OH adsorption with reasonable accuracy, which is critical for understanding the performance of catalysts in HER.


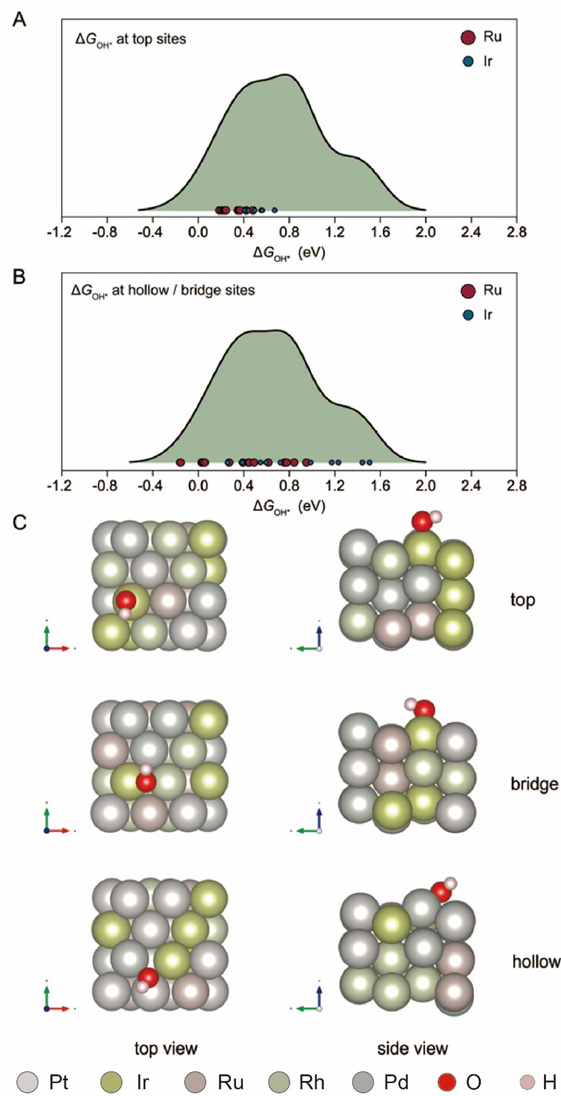


**Figure S25.** OH-adsorption behaviors on selected sites, and OH-adsorption configurations of RuRhPdPtIr HEA layers. (A) ∆*G*_OH*_ at top sites where Ru and Ir are present. (B) ∆*G*_OH*_ at bridge and hollow sites where Ru and Ir are present. (C) OH-adsorption configurations of RuRhPdPtIr hcp (100) surface. In the RuRhPdPtIr hcp (100) surface, Ir shows optimal OH-adsorption free energy as compared to Ru in top, hollow, and bridge sites ((A) and (B)). They align with *operando* XAS measurements obtained in this study, where Ir is identified as an electron-donating site (Figure 7C), enabling efficient conversion of OH_ad_ to OH⁻ (OH_ad_ + e⁻ → OH⁻).

**Table S1.** Compositions, synthetic methods, crystal structures, and catalytic applications of various HEA nanocrystals in recent reports.

| Compositions | Synthetic methods | Crystal structures | Catalytic applications | Ref. |
| --- | --- | --- | --- | --- |
| PtPdCoNiFeCuAuSn | Carbothermal shock | FCC | Ammonia oxidation | ^[13]^ |
| PtIrCuNiCr | Laser scanning ablation | FCC | HER and OER (oxygen evolution reaction) | ^[14]^ |
| RuRhPdAgOsIrPtAu | Wet-chemical synthesis | FCC | HER | ^[15]^ |
| PdPtRhIrRu | Dropwise synthesis | FCC | HER and HOR (hydrogen oxidation reaction) | ^[16]^ |
| RuIrFeCoNi | Droplet-to-particle | FCC | ORR (oxygen reduction reaction) and OER | ^[17]^ |
| PtNiFeCoCu | Wet-chemical synthesis | FCC | HER and MOR (methanol oxidation reaction) | ^[18]^ |
| PtRuNiCoFeMo | Wet-chemical synthesis | FCC | HOR | ^[19]^ |
| PtPdFeCoNi | Microwave heating | FCC | -- | ^[20]^ |
| PtZrNbFeCuTaMoHfBiWZnSnPdNi | Step-alloying | FCC | HER, ORR and MOR | ^[21]^ |
| CoNiCuRuPd | Hydrogen spillover-driven synthesis | FCC | CO_2_ hydrogenation | ^[22]^ |
| IrPdPtRhRu | Continuous-flow reactor synthesis | FCC | HER | ^[23]^ |
| CoNiCuMnMo | Pyrolysis reduction | FCC | GOR (glycerol oxidation reaction) | ^[24]^ |
| PdMoGaInNi | Wet-chemical synthesis | FCC | HER | ^[25]^ |
| FeCoNiMoW | Wet-chemical synthesis | FCC | OER and ORR | ^[26]^ |
| PdNiRuIrRh | Wet-chemical synthesis | FCC | HOR | ^[27]^ |
| RuRhPdOsIrPt | Wet-chemical synthesis | FCC | EOR (ethanol oxidation reaction) | ^[28]^ |
| PtCoCuRuNiFe | Spray drying technique and thermal decomposition reduction | FCC | MOR | ^[29]^ |
| FeCoNiCuMn | Solvothermal synthesis | FCC | CO_2_ reduction | ^[30]^ |
| RuRhCoNiIr | High-temperature synthesis | FCC | NH_3_ decomposition | ^[31]^ |
| CoMoFeNiCu | Carbothermal shock | FCC | NH_3_ decomposition | ^[32]^ |
| PtFeCoNiCu | Solvothermal reaction and thermal annealing | FCC | HER | ^[33]^ |
| CrFeCoNiMo | Thermal plasma synthesis | FCC | Photothermal conversion | ^[34]^ |
| MnCoNiCuRhPdSnIrPtAu | Fast moving bed pyrolysis | FCC | -- | ^[35]^ |
| FeCoNiMnRu | Electrospinning and graphitization | FCC | HER and OER | ^[36]^ |
| PtPdCuNiCo | Hydrogen reduction | FCC | EOR | ^[37]^ |
| AuAgPtPdCu | Melting and cryogrinding | FCC | CO_2_ reduction | ^[38]^ |
| PtCoNiRuIr | High-temperature liquid shock | FCC | HER | ^[39]^ |
| PtFeCoNiMn | Shock heating and shock cooling | FCC | ORR | ^[40]^ |
| NiCoFePtRh | Chemical coreduction and annealing | FCC | HER | ^[41]^ |
| IrRuRhMoW | Rapid co-decomposition | FCC | HOR and HER | ^[42]^ |

**Table S2.** Estimation of the average number (n) of RuRhPdPtIr atomic layers on the Ru seeds via ICP-OES results. For the Ru@RuRhPdPtIr core-shell nanocrystals, Table S2 presents the ICP-OES data derived from the same sample shown in Figure 1J.

|  | Atomic ratio (at.%) of the elements in Ru@RuRhPdPtIr core-shell nanocrystals | | | | | |
| --- | --- | --- | --- | --- | --- | --- |
| Sample | core | shell | | | | |
|  | Ru | Ru | Rh | Pd | Pt | Ir |
| Ru@RuRhPdPtIr | 51.2 | 9.8 | 9.1 | 9.5 | 9.9 | 10.5 |

We assume that the Ru seeds are spherical and maintain their shape after the epitaxial growth of HEA atomic layers. By knowing the volume of the seed and the atomic ratio of the core in the core-shell nanocrystals, we can determine the volume of the entire core-shell nanocrystal. The volume of nanocrystals is calculated by the following equation:

$The volume of nanocrystals= \frac{4}{3}\times\pi\times{(\frac{d}{2})}^{3}$ (2)

where d is the diameter of the nanocrystals. The average diameter of Ru seeds is 6.70 nm (Figure S1), resulting in a calculated volume of 157.5 nm^3^. The volume of core-shell nanocrystals can be calculated by the following equation:

$The volume of core\text{-}shell nanocrystals= \frac{the volume of seeds}{the atomic ratio of core}$ (3)

Then, we can extrapolate the diameter of core-shell nanocrystals:

$$The diameter of core\text{-}shell nanocrystals$$

$=2\times{(the volume of core\text{-}shell nanocrystals\times\frac{3}{4\pi})}^{1/3}$ (4)

The diameter of Ru@RuRhPdPtIr core-shell nanocrystals is extrapolated to be 8.38 nm based on the aforementioned ICP-OES data. Then, the number of atomic layers can be calculated using the following formula:

$$The number of atomic layers$$

$= \frac{1}{2}\times\frac{(the diameter of core\text{-}shell nanocrystals-the diameter of seeds)}{lattice spacing}$ (5)

where the lattice spacing of Ru@RuRhPdPtIr is set to be 0.19 nm according to the HAADF-STEM image in Figure 1E. The number of RuRhPdPtIr atomic layers on Ru seeds is estimated to be around 4.4, based on the aforementioned calculations. This estimation is consistent with the TEM observations (Figure 1D), which show that the total thickness of the HEA shells is approximately 1.7 nm. With each side contributing about 0.85 nm, this corresponds to around 4 atomic layers.

**Table S3.** The binding energies of all the elements in Ru@Ru_0.2_Rh_0.2_Pd_0.2_Pt_0.2_Ir_0.2-4L_ core−shell nanocrystals determined by XPS analysis.

| Chemical state | Ru 3d_5/2_ | Rh 3d_5/2_ | Pd 3d_5/2_ | Pt 4f_7/2_ | Ir 4f_7/2_ |
| --- | --- | --- | --- | --- | --- |
| Table lookup value^[43]^ | 280.1 | 307.2 | 335.1 | 71.2 | 60.9 |
| Ru@Ru_0.2_Rh_0.2_Pd_0.2_Pt_0.2_Ir_0.2-4L_ core−shell nanocrystals | 280.1 | 307.4  (peak value) | 335.5 | 71.3 | 60.7 |

**Table S4.** The lattice mismatch between Ru core and RuRhPdPtIr shell.

| Lattice parameter in HCP structure | | | | | |
| --- | --- | --- | --- | --- | --- |
| Element | Ru | Rh | Pd | Pt | Ir |
| *a* (Å) | 2.71 | 2.73 | 2.78 | 2.76 | 2.75 |
| *b* (Å) | 2.71 | 2.73 | 2.78 | 2.76 | 2.75 |
| *c* (Å) | 4.27 | 4.43 | 4.63 | 4.79 | 4.46 |

We calculate the lattice parameter of RuRhPdPtIr shell in HCP structure according to the following formula^[44]^:

$${lattice parameter}_{weighted average}$$

$=\sum_{i=1}^{5} (\% of i^{th} element present \times lattice parameter of i^{th} element)$ (6)

|  | HCP-Ru_0.2_Rh_0.2_Pd_0.2_Pt_0.2_Ir_0.2_ |
| --- | --- |
| *a* (Å) | 2.746 |
| *b* (Å) | 2.746 |
| *c* (Å) | 4.516 |

The lattice mismatch between Ru core and RuRhPdPtIr shell is derived from the following equation^[45]^:

$lattice mismatch=\left| \frac{shell lattice parameter -core lattice parameter}{core lattice parameter} \right|$ (7)

| core | HCP-Ru |
| --- | --- |
| shell | HCP-Ru_0.2_Rh_0.2_Pd_0.2_Pt_0.2_Ir_0.2_ |
| Lattice mismatch on *a* axis (%) | 1.33 |
| Lattice mismatch on *c* axis (%) | 5.76 |

**Table S5.** The lattice mismatch between Pd core and RuRhPdPtIr shell.

| Lattice parameter in FCC structure | | | | | |
| --- | --- | --- | --- | --- | --- |
| Element | Ru | Rh | Pd | Pt | Ir |
| *a* (Å) | 3.79 | 3.81 | 3.92 | 3.94 | 3.85 |
| *b* (Å) | 3.79 | 3.81 | 3.92 | 3.94 | 3.85 |
| *c* (Å) | 3.79 | 3.81 | 3.92 | 3.94 | 3.85 |

We calculate the lattice parameter of RuRhPdPtIr shell in the FCC structure according to the formula (6) in Table S4. The calculated lattice parameters along three axes are presented below:

|  | FCC- Ru_0.2_Rh_0.2_Pd_0.2_Pt_0.2_Ir_0.2_ |
| --- | --- |
| *a* (Å) | 3.862 |
| *b* (Å) | 3.862 |
| *c* (Å) | 3.862 |

The lattice mismatch between Pd core and RuRhPdPtIr shell is derived from the equation (7) in Table S4. The calculated results of the lattice mismatch in three axes are shown below:

| core | FCC-Pd |
| --- | --- |
| shell | FCC- Ru_0.2_Rh_0.2_Pd_0.2_Pt_0.2_Ir_0.2_ |
| Lattice mismatch on *a, b,* and *c* axis (%) | 1.50 |

**Video S1.** Three-dimensional (3D) EDS tomography of Ru@Ru_0.2_Rh_0.2_Pd_0.2_Pt_0.2_Ir_0.2-4L_ core-shell nanocrystals.

**References**

[1] A. Janssen, V. Pawlik, A. D. von Rueden, L. Xu, C. Wang, M. Mavrikakis, Y. Xia, *Adv. Mater.* **2021**, *33*, 2103801.

[2] C. J. Hsieh, Y. H. Liu, C. Y. Tsao, J. T. Lin, C. C. Chi, C. W. Chang, Y. C. Hsiao, C. Y. Wu, T. H. Yang, *Adv. Mater. Interfaces* **2022**, *9*, 2201036.

[3] Q. Hu, K. Gao, X. Wang, H. Zheng, J. Cao, L. Mi, Q. Huo, H. Yang, J. Liu, C. He, *Nat. Commun.* **2022**, *13*, 3958.

[4] D. M. Morales, M. Risch, *JPhys Energy* **2021**, *3*, 034013.

[5] J. Chen, Y. M. Yiu, Z. Wang, D. Covelli, R. Sammynaiken, Y. Z. Finfrock, T.-K. Sham, *J. Phys. Chem. C* **2020**, *124*, 2313-2318.

[6] M. Łukaszewski, M. Soszko, A. Czerwiński, *Int. J. Electrochem. Sci.* **2016**, *11*, 4442-4469.

[7] Y. Li, L. A. Zhang, Y. Qin, F. Chu, Y. Kong, Y. Tao, Y. Li, Y. Bu, D. Ding, M. Liu, *ACS. Catal.* **2018**, *8*, 5714-5720.

[8] Z. Zhang, H. Liu, L. Ni, Z. L. Zhao, H. Li, *J. Energy Chem*. **2022**, *72*, 176-185.

[9] T. A. A. Batchelor, J. H. Pedersen, S. H. Winther, I. E. Castelli, K. W. Jacobsen, J. Rossmeisl, *Joule* **2019**, *3*, 834-845.

[10] J. K. Norskov, T. Bligaard, A. Logadottir, J. R. Kitchin, J. G. Chen, S. Pandelov, U. Stimming, *J. Electrochem. Soc.* **2005**, *152*, J23.

[11] J. Greeley, T. F. Jaramillo, J. Bonde, I. Chorkendorff, J. K. Norskov, *Nat. Mater.* **2006**, *5*, 909-913.

[12] I. C. Man, H. Y. Su, F. Calle-Vallejo, H. A. Hansen, J. I. Martínez, N. G. Inoglu, J. Kitchin, T. F. Jaramillo, J. K. Nørskov, J. Rossmeisl, *Chemcatchem.* **2011**, *3*, 1559-1165.

[13] Y. Yao, Z. Huang, P. Xie, S. D. Lacey, R. J. Jacob, H. Xie, F. Chen, A. Nie, T. Pu, M. Rehwoldt, *Science* **2018**, *359*, 1489-1494.

[14] B. Wang, C. Wang, X. Yu, Y. Cao, L. Gao, C. Wu, Y. Yao, Z. Lin, Z. Zou, *Nat. Synth.* **2022**, *1*, 138-146.

[15] D. Wu, K. Kusada, Y. Nanba, M. Koyama, T. Yamamoto, T. Toriyama, S. Matsumura, O. Seo, I. Gueye, J. Kim, *J. Am. Chem. Soc.* **2022**, *144*, 3365-3369.

[16] Y.-H. Liu, C.-J. Hsieh, L.-C. Hsu, K.-H. Lin, Y.-C. Hsiao, C.-C. Chi, J.-T. Lin, C.-W. Chang, S.-C. Lin, C.-Y. Wu, *Sci. Adv.* **2023**, *9*, eadf9931.

[17] X. Wang, Q. Dong, H. Qiao, Z. Huang, M. T. Saray, G. Zhong, Z. Lin, M. Cui, A. Brozena, M. Hong, *Adv. Mater.* **2020**, *32*, 2002853.

[18] H. Li, Y. Han, H. Zhao, W. Qi, D. Zhang, Y. Yu, W. Cai, S. Li, J. Lai, B. Huang, *Nat. Commun.* **2020**, *11*, 5437.

[19] C. Zhan, Y. Xu, L. Bu, H. Zhu, Y. Feng, T. Yang, Y. Zhang, Z. Yang, B. Huang, Q. Shao, *Nat. Commun.* **2021**, *12*, 6261.

[20] H. Qiao, M. T. Saray, X. Wang, S. Xu, G. Chen, Z. Huang, C. Chen, G. Zhong, Q. Dong, M. Hong, *ACS Nano* **2021**, *15*, 14928-14937.

[21] Y. Wang, W. Luo, S. Gong, L. Luo, Y. Li, Y. Zhao, Z. Li, *Adv. Mater.* **2023**, *35*, 2302499.

[22] K. Mori, N. Hashimoto, N. Kamiuchi, H. Yoshida, H. Kobayashi, H. Yamashita, *Nat. Commun.* **2021**, *12*, 3884.

[23] H. Minamihara, K. Kusada, D. Wu, T. Yamamoto, T. Toriyama, S. Matsumura, L. S. R. Kumara, K. Ohara, O. Sakata, S. Kawaguchi, *J. Am. Chem. Soc.* **2022**, *144*, 11525-11529.

[24] L. Fan, Y. Ji, G. Wang, J. Chen, K. Chen, X. Liu, Z. Wen, *J. Am. Chem. Soc.* **2022**, *144*, 7224-7235.

[25] X. Fu, J. Zhang, S. Zhan, F. Xia, C. Wang, D. Ma, Q. Yue, J. Wu, Y. Kang, *ACS Catal.* **2022**, *12*, 11955-11959.

[26] R. He, L. Yang, Y. Zhang, D. Jiang, S. Lee, S. Horta, Z. Liang, X. Lu, A. Ostovari Moghaddam, J. Li, *Adv. Mater.* **2023**, *35*, 2303719.

[27] Y. Men, D. Wu, Y. Hu, L. Li, P. Li, S. Jia, J. Wang, G. Cheng, S. Chen, W. Luo, *Angew. Chem. Int. Ed.* **2023**, *62*, e202217976.

[28] D. Wu, K. Kusada, T. Yamamoto, T. Toriyama, S. Matsumura, S. Kawaguchi, Y. Kubota, H. Kitagawa, *J. Am. Chem. Soc.* **2020**, *142*, 13833-13838.

[29] P. Zhao, Q. Cao, W. Yi, X. Hao, J. Li, B. Zhang, L. Huang, Y. Huang, Y. Jiang, B. Xu, *ACS Nano* **2022**, *16*, 14017-14028.

[30] H. Huang, J. Zhao, H. Guo, B. Weng, H. Zhang, R. A. Saha, M. Zhang, F. Lai, Y. Zhou, R. Z. Juan, *Adv. Mater.* **2024**, 2313209.

[31] Y. Yao, Z. Liu, P. Xie, Z. Huang, T. Li, D. Morris, Z. Finfrock, J. Zhou, M. Jiao, J. Gao, *Sci. Adv.* **2020**, *6*, eaaz0510.

[32] P. Xie, Y. Yao, Z. Huang, Z. Liu, J. Zhang, T. Li, G. Wang, R. Shahbazian-Yassar, L. Hu, C. Wang, *Nat. Commun.* **2019**, *10*, 4011.

[33] Z. W. Chen, J. Li, P. Ou, J. E. Huang, Z. Wen, L. Chen, X. Yao, G. Cai, C. C. Yang, C. V. Singh, *Nat. Commun.* **2024**, *15*, 359.

[34] K. S. Kim, M. Couillard, Z. Tang, H. Shin, D. Poitras, C. Cheng, O. Naboka, D. Ruth, M. Plunkett, L. Chen, *Nat. Commun.* **2024**, *15*, 1450.

[35] S. Gao, S. Hao, Z. Huang, Y. Yuan, S. Han, L. Lei, X. Zhang, R. Shahbazian-Yassar, J. Lu, *Nat. Commun.* **2020**, *11*, 2016.

[36] J. Hao, Z. Zhuang, K. Cao, G. Gao, C. Wang, F. Lai, S. Lu, P. Ma, W. Dong, T. Liu, *Nat. Commun.* **2022**, *13*, 2662.

[37] W. Peng, Y.-R. Lu, H. Lin, M. Peng, T.-S. Chan, A. Pan, Y. Tan, *ACS Nano* **2023**, *17*, 22691-22700.

[38] S. Nellaiappan, N. K. Katiyar, R. Kumar, A. Parui, K. D. Malviya, K. Pradeep, A. K. Singh, S. Sharma, C. S. Tiwary, K. Biswas, *ACS Catal.* **2020**, *10*, 3658-3663.

[39] X. Cui, Y. Liu, X. Wang, X. Tian, Y. Wang, G. Zhang, T. Liu, J. Ding, W. Hu, Y. Chen, *ACS Nano* **2024**, *18*, 2948-2957.

[40] K. Wang, R. Chen, H. Yang, Y. Chen, H. Jia, Y. He, S. Song, Y. Wang, *Adv. Funct. Mater.* **2024**, *34*, 2310683.

[41] G. Feng, F. Ning, J. Song, H. Shang, K. Zhang, Z. Ding, P. Gao, W. Chu, D. Xia,  *J. Am. Chem. Soc.* **2021**, *143*, 17117-17127.

[42] H. Luo, L. Li, F. Lin, Q. Zhang, K. Wang, D. Wang, L. Gu, M. Luo, F. Lv, S. Guo, *Adv. Mater.* **2024**, *36*, 2403674.

[43] J. F. Modulder, W. F. Stickle, P. E. Sobol, K. D. Bomben, *Handbook of X-ray photoelectron spectroscopy*, Perkin-Elmer Corporation, **1992**.

[44] G. R. Dey, C. R. McCormick, S. S. Soliman, A. J. Darling, R. E. Schaak, *ACS Nano* **2023**, *17*, 5943-5955.

[45] Y. Xia, K. D. Gilroy, H. C. Peng, X. Xia, *Angew. Chem. Int. Ed.* **2017**, *56*, 60-95.
